# Challenges of Telemonitoring Programs for Complex Chronic Conditions: Randomized Controlled Trial With an Embedded Qualitative Study

**DOI:** 10.2196/31754

**Published:** 2022-01-26

**Authors:** Patrick Ware, Amika Shah, Heather Joan Ross, Alexander Gordon Logan, Phillip Segal, Joseph Antony Cafazzo, Katarzyna Szacun-Shimizu, Myles Resnick, Tessy Vattaparambil, Emily Seto

**Affiliations:** 1 Centre for Global eHealth Innovation University Health Network Toronto, ON Canada; 2 Institute of Health Policy, Management and Evaluation Dalla Lana School of Public Health University of Toronto Toronto, ON Canada; 3 Peter Munk Cardiac Centre University Health Network Toronto, ON Canada; 4 Ted Rogers Centre for Heart Research University Health Network Toronto, ON Canada; 5 Lunenfeld-Tanenbaum Research Institute Mount Sinai Hospital Toronto, ON Canada; 6 Division of Nephrology Mount Sinai Hospital Toronto, ON Canada; 7 Division of Nephrology University Health Network Toronto, ON Canada; 8 Division of Endocrinology University Health Network Toronto, ON Canada; 9 Department of Medicine University of Toronto Toronto, ON Canada; 10 Institute of Biomaterials and Biomedical Engineering University of Toronto Toronto, ON Canada

**Keywords:** telemonitoring, telemedicine, heart failure, diabetes, hypertension, tertiary health care, multiple chronic conditions, mobile phone

## Abstract

**Background:**

Despite the growing prevalence of people with complex conditions and evidence of the positive impact of telemonitoring for single conditions, little research exists on telemonitoring for this population.

**Objective:**

This randomized controlled trial and embedded qualitative study aims to evaluate the impact on and experiences of patients and health care providers (HCPs) using a telemonitoring system with decision support to manage patients with complex conditions, including those with multiple chronic conditions, compared with the standard of care.

**Methods:**

A pragmatic, unblinded, 6-month randomized controlled trial sought to recruit 146 patients with ≥1 diagnosis of heart failure (HF), uncontrolled hypertension (HT), and insulin-requiring diabetes mellitus (DM) from outpatient specialty settings in Toronto, Ontario, Canada. Participants were randomized into the control and telemonitoring groups, with the latter being instructed to take readings relevant to their conditions. The telemonitoring system contained an algorithm that generated decision support in the form of actionable self-care directives to patients and alerts to HCPs. The primary outcome was health status (36-Item Short Form Health Survey questionnaire). Secondary outcomes included anxiety and depression, self-efficacy in chronic disease management, and self-reported health service use. HF-related quality of life and self-care measures were also collected from patients followed for HF. Within- and between-group change scores were analyzed for statistical significance (*P*<.05). A convenience sample of HCPs and patients in the intervention group was interviewed about their experiences.

**Results:**

A total of 96 patients were recruited and randomized. Recruitment was terminated early because of implementation challenges and the onset of the COVID-19 pandemic. No significant within- and between-group differences were found for the main primary and secondary outcomes. However, a within-group analysis of patients with HF found improvements in self-care maintenance (*P*=.04) and physical quality of life (*P*=.046). Opinions expressed by the 5 HCPs and 13 patients who were interviewed differed based on the monitored conditions. Although patients with HF reported benefitting from actionable self-care guidance and meaningful interactions with their HCPs, patient and HCP users of the DM and HT modules did not think telemonitoring improved the clinical management of those conditions to the same degree. These differing experiences were largely attributed to the siloed nature of specialty care and the design of the decision support, whereby fluctuations in the status of HT and DM typically required less urgent interventions compared with patients with HF.

**Conclusions:**

We recommend that future research conceive telemonitoring as a *program* and that self-management and clinical decision support are necessary but not sufficient components of such programs for patients with complex conditions and lower acuity. We conclude that telemonitoring for patients with complex conditions or within multidisciplinary care settings may be best operationalized through nurse-led models of care.

**Trial Registration:**

ClinicalTrials.gov NCT03127852; https://clinicaltrials.gov/ct2/show/NCT03127852

**International Registered Report Identifier (IRRID):**

RR2-10.2196/resprot.8367

## Introduction

### Telemonitoring for the Management of Chronic Conditions

Despite a growing prevalence of patients with complex and multiple chronic conditions (MCCs) [1,2], siloed care models focusing on single conditions have been a barrier to the appropriate management of these patients [3]. In Canada, 12.9% of individuals across all age groups report having ≥2 chronic conditions, and 3.9% report having ≥3 conditions [1]. They are among the highest cost users of health care systems because of a higher frequency of hospitalizations, many of which are thought to be preventable [4,5]. Research suggests that effective patient self-management can reduce the need for urgent care while promoting self-efficacy, improving quality of life, and reducing the risk of adverse psychological effects [6]. However, complex decision-making and often conflicting clinical advice from multiple siloed health care providers (HCPs) make self-management challenging for patients with MCCs [7,8].

Telemonitoring has the potential to empower patients and HCPs by facilitating patient self-management and clinical decision support to manage MCCs. Telemonitoring systems enable patients to track vital signs and symptoms and can enable the automatic generation of self-management instructions [9]. In addition, by delivering these data to the clinical team, HCPs can identify patients showing early signs of exacerbation, which offers an opportunity for reinforcing the principles of self-management at *teachable moments* [10]. Importantly, telemonitoring allows HCPs to provide remote guidance or make changes to a care plan, thereby stabilizing symptoms before they escalate to the point of hospitalization.

Although research on telemonitoring is rapidly growing [11], these systems typically target a singular condition such as diabetes mellitus (DM), hypertension (HT), or heart failure (HF) [12-14]. Systematic reviews indicate that telemonitoring for single conditions leads to improved health outcomes and quality of life and reductions in health service use and costs [15-21]. Studies that do not report improvements do not often include a self-care component, are difficult to use, or do not target patients who are most ill and frequently hospitalized [22-25]. To date, few studies have focused on the use of telemonitoring among patients with complex conditions, although these patients may benefit the most from such interventions [15,26-32].

### Objective

The objective of this study is to evaluate the impact of a mobile phone–based telemonitoring program for the management of patients with complex conditions in specialty care settings. Patients with complex conditions are defined as those who are at high risk for hospitalization, exacerbations of their chronic conditions, and disease progression and those with MCCs, including HF, uncontrolled HT, and insulin-requiring DM. The primary research question was the following: *what is the impact of a telemonitoring program for patients with complex conditions on health status, self-management, and health service use?* The secondary research question was the following: *what were the experiences of patients and HCPs related to the telemonitoring program and the way in which it was implemented?*

## Methods

### Study Design and Setting

This was a pragmatic, unblinded, 1:1 randomized controlled trial (RCT) comparing the 6-month impact of telemonitoring to support the management of patients with complex conditions with that of the standard of care. An embedded qualitative component was included to understand the results of the trial. Patients with HF were recruited from the Heart Function Clinic at the University Health Network (UHN), a large academic hospital in Toronto, Ontario, Canada, between August 2016 and February 2018. Patients with HT were recruited from an HT clinic at Mount Sinai Hospital between July 2019 and December 2019, and patients with DM were recruited from the UHN Endocrinology Clinic between August 2019 and December 2019. The study received approval from the UHN (15-9995-BE) and Mount Sinai Hospital (16-0093-E) research ethics boards, which approved the procedures to ensure patient privacy, including anonymization of data during storage and analysis.

### Participants

To be eligible, patients had to be aged ≥18 years; able to speak and read English (or have a caregiver who does); and diagnosed with HF with reduced ejection fraction (<40%), uncontrolled HT (≥140/90 mm Hg auscultatory), or insulin-requiring DM and performing self-capillary glucose monitoring. Exclusion criteria included being on mechanical circulatory support, dialysis, or a transplant list. In addition, patients with a life expectancy <1 year, dementia, uncontrolled psychiatric illness, or residents of a long-term care facility were excluded. Refer to the published protocol for the full criteria [33].

### Treatment Arms

#### Telemonitoring Group

**Figure 1:**
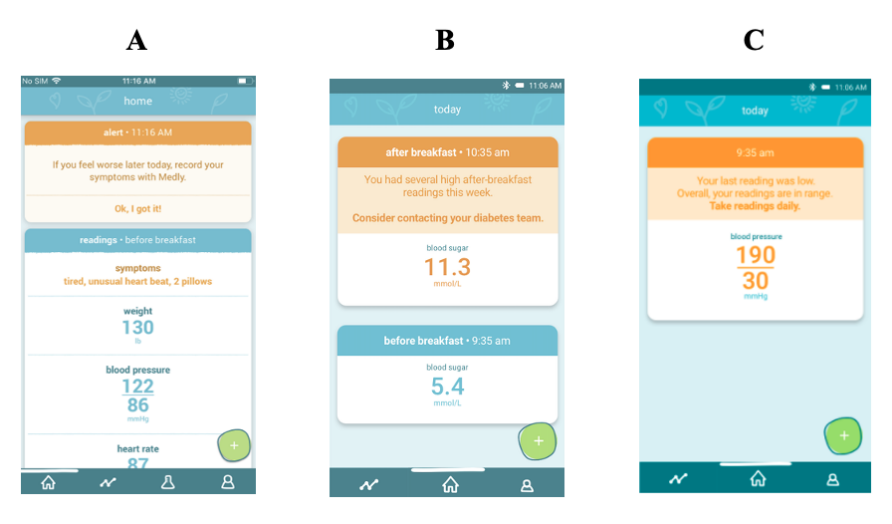
Screens of the Medly multiple chronic conditions app showing self-care feedback for (A) heart failure, (B) diabetes mellitus, and (C) hypertension.

The intervention was a mobile phone–based telemonitoring program involving a system named Medly (UHN), which enables patients with chronic conditions to take relevant physiological measurements with wireless home medical devices and answer symptom questions using the Medly smartphone app. In response to these inputs, rule-based algorithms, which were iteratively developed and validated by HF, DM, and HT specialists [34] and customized through target thresholds, displayed self-care instructions to patients (Figure 1) and sent alerts to the clinical team via email and a secure web portal where historical trends could also be viewed [34]. As such, the system was designed to improve patient self-management and provide clinical decision support to HCPs [35]. Participants were provided with the necessary equipment, including a smartphone and relevant Bluetooth devices (weight scale, blood pressure monitor, and blood glucose monitor) [33].

Patients with HF were instructed to monitor their daily weight, blood pressure, heart rate, and symptoms. Owing to the frequency of readings and higher complexity of the HF Medly algorithm [36], patient feedback was designed to be highly actionable. For example, patients were told to take a dose of their prescribed diuretic and restrict salts and fluids upon a large weight gain. Patients with DM were instructed to record blood glucose readings at least once per week or more as instructed by their HCP and received actionable feedback (eg, “Eat 15 g [1 tbls] of sugar or other fast-acting carbohydrates” in response to low blood glucose readings). Patients with HT were instructed to report their blood pressure once every 2 weeks unless the readings were out of range, in which case, they would be instructed through the app to increase the frequency of their readings. All modules had messages instructing the users to contact the clinic or go to the emergency department (ED) when critical parameters were out of range. To assist with adherence, an automated phone call was sent to patients based on the required frequency of each condition’s algorithm.

Although the intent was for patients with MCCs to be followed holistically, development delays for the HT and DM modules led to patients with HF being enrolled and managed for HF alone, even if they had MCCs. In addition, the siloed nature of specialty care made it such that even when the technology could support the simultaneous management of HF, DM, and HT, patients were only monitored for the condition being managed at the location of enrollment. As a result, the model of care differed depending on the structure and resources at each clinic. For example, alerts for patients with HF were primarily addressed by nurse practitioners who would escalate issues to the treating cardiologist as required. In contrast, telemonitoring alert management for HT and DM was the responsibility of the treating physician.

#### Standard of Care Group

Standard of care followed Canadian clinical care guidelines for HF, DM, and HT [37-39]. In general, that included seeing the clinical team for scheduled follow-ups every 3 to 6 months, optimization of medical therapy, and self-management education.

### Enrollment and Randomization

A total of 146 patients with varying chronic conditions were targeted for enrollment (see sample size justification [33]). HCPs familiar with the study’s inclusion criteria identified patients during scheduled outpatient appointments and introduced them to an on-site research coordinator. After confirming eligibility, the coordinator explained the study and obtained informed consent. The patients were then block randomized using blocks of 4 into the intervention and standard of care treatment groups in a 1:1 ratio as per the published stratification protocol [33]. Patients allocated to the intervention arm were provided with the telemonitoring equipment and user manual before receiving face-to-face training on how to use the equipment.

### Outcomes

The primary outcome was health status as measured by the 36-Item Short Form Health Survey questionnaire. Secondary outcomes included anxiety and depression, as measured using the Hospital Anxiety and Depression Scale [40], and patients’ self-efficacy to manage their condition, as measured using the Self-Efficacy for Managing Chronic Disease 6-Item scale [41-43]; the number of self-reported interactions with the health system in the previous 6 months was also collected, including hospitalizations and visits to ED, specialty care clinics, and family physicians. Self-care, as measured by the Self-Care of HF Index [44], and HF-specific quality of life, as measured by the Minnesota Living with HF Questionnaire, [45] were also collected for patients with HF.

### Quantitative Data Collection and Analysis

Questionnaires containing the patient-reported outcome measures were administered at baseline and at 6 months [33]. Demographic questions were included in the baseline questionnaire to characterize the study participants. Patient adherence was calculated from the Medly server log data as a percentage of the completed recommended readings over the course of the 6-month trial.

Posttrial data and change scores were compared between the treatment arms using independent Student *t* tests and Mann–Whitney tests (for normally and not normally distributed data, respectively). Paired Student *t* tests and Wilcoxon signed-rank tests were performed to compare baseline and poststudy data within the control and telemonitoring groups. Analyses were performed using SPSS (version 27; IBM Corp) under the intention-to-treat principle and using a significance of *P*<.05.

### Qualitative Data Collection and Analysis

All HCPs who used the system and a sample of patients in the intervention group were invited to participate in poststudy semistructured interviews. The interviews began with open-ended questions about the participants’ experiences with the intervention and had probing questions inspired by the constructs of performance expectancy, effort expectancy, social influence, and facilitating conditions from the unified theory of acceptance and use of technology [46]. Patients were identified through a convenience sample with efforts made to include patients from the 3 monitored conditions. Interviews took place in a private clinic room or over the telephone and were audio recorded for later transcription.

Qualitative data were analyzed using a conventional content analysis approach [47] by 2 researchers (AS and PW). An initial round of independent open coding was conducted with the primary objective of organizing quotes into themes that explained the quantitative results. Then, AS and PW met to discuss themes and agree upon a coding framework that was applied in the second round of deductive coding. A final discussion was held to review the results and reach a consensus. NVivo (QSR International; version 11) was used to help organize the source documents and themes.

## Results

### Study Participants

**Figure 2:**
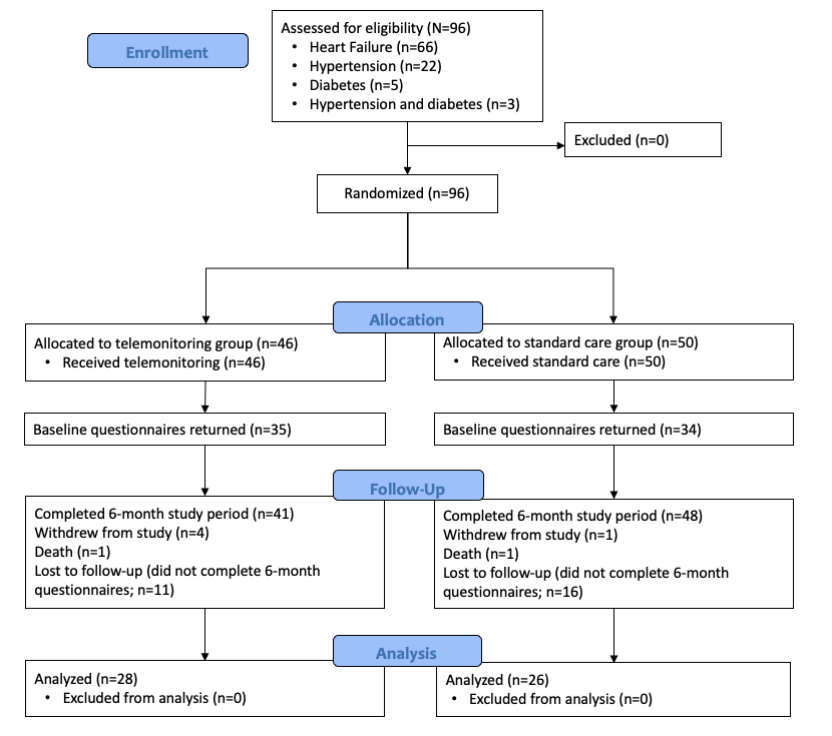
Flow of patient participants through the trial.

Recruitment ended after the enrollment of 96 patients, of which 66 (69%) were followed for HF, 22 (23%) were followed for uncontrolled HT, 5 (5%) were followed for insulin-requiring DM, and 3 (3%) were followed for both HT and DM (Figure 2). The decision to stop recruitment before reaching the target sample size was made for 3 key reasons. First, the HF telemonitoring program became standard of care at the UHN Heart Function clinic, meaning there would no longer be a difference between the treatment arms. Second, recruitment challenges were observed for patients with DM because of the rapid emergence and growing use of continuous and flash glucose monitors, which meant that few patients met the criteria of self-capillary glucose monitoring. Finally, and most importantly, the COVID-19 pandemic led to a pause of nonessential research activities and a significant shift toward virtual care, which fundamentally altered the control group after the research pause was lifted.

In addition to ending recruitment, shifting priorities at the onset of the COVID-19 pandemic also affected the collection of poststudy data, as patients followed for HT and DM ended their enrollment during the first wave, which led to a higher rate of incomplete questionnaires. A death was reported in each treatment arm; however, these were not considered as adverse events of the study. Therefore, although 45 and 50 patients were allocated to the telemonitoring and control arms, only 29% (28/96) and 27% (26/96) of complete data sets were available for analysis in the telemonitoring and control arms, respectively. Study participants were predominantly male (54/96, 56%) and had an average age of 59 (SD 12.6) years. These, along with the other demographics presented in Table 1, are representative of the UHN Heart Function Clinic, from which most patient participants were recruited.

**Table 1:** Baseline characteristics of patient participants (N=96).

Characteristics			Telemonitoring group (n=46)		Control group (n=50)		Total
Age (years), mean (SD)			62 (12.6)		55 (14.3)		59 (13.9)
**Sex, n (%)**							
	Male	28 (61)		26 (52)		54 (56)	
	Female	9 (20)		8 (16)		17 (18)	
**Ethnicity, n (%)**							
	White	22 (48)		24 (48)		46 (48)	
	Black	2 (4)		4 (8)		6 (6)	
	Asian	3 (7)		6 (12)		9 (9)	
	Other	7 (15)		2 (4)		9 (9)	
**Rurality, n (%)**							
	Urban	20 (43)		23 (46)		43 (45)	
	Suburban	11 (24)		11 (22)		22 (23)	
	Rural	2 (4)		2 (4)		2 (2)	
**Highest education achieved, n (%)**							
	Less than high school	4 (9)		1 (2)		5 (5)	
	High school	5 (11)		4 (8)		9 (9)	
	Trade or technical training	5 (11)		6 (12)		11 (11)	
	College or university	15 (33)		19 (38)		34 (35)	
	Postgraduate	5 (11)		5 (10)		10 (10)	
**Comfort with smartphone, n (%)**							
	Not comfortable	1 (2)		0 (0)		1 (1)	
	Somewhat comfortable	8 (17)		4 (8)		12 (13)	
	Comfortable	8 (17)		28 (56)		16 (17)	
	Very comfortable	13 (28)		15 (30)		28 (29)	

### Quantitative Outcomes

Table 2 shows the results from the statistical analyses. The within-group pre–post comparisons, poststudy between-group comparisons, and between-group change score comparisons revealed no significant differences in the primary outcome of health status as measured by the 36-Item Short Form Health Survey questionnaire physical and emotional subscales. Similarly, no statistically significant differences were observed for any of the secondary outcomes relevant to the entire sample, including anxiety and depression (as measured by the Hospital Anxiety and Depression Scale), self-efficacy (as measured by the Self-Efficacy for Managing Chronic Disease 6-Item scale), and self-reported use metric. An exception was a reduction in self-reported hospitalizations across all patients, which was significant in the control group.

**Table 2:** Independent Student *t* test^a^ for SF-36^b^, HADS^c^, SEMCD6^d^, self-reported use, MLHFQ^e^, and SCHFI^f^.

Parameter			Telemonitoring group (n=46)				Standard care group (n=50)					Between-group poststudy data, *P* value		Between-group change scores, *P* value
			Values, n (%)	Baseline, mean (SD)	Poststudy, mean (SD)	*P* value	Values, n (%)	Baseline, mean (SD)	Poststudy, mean (SD)	*P* value				
	**SF-36**													
		Physical component	24 (52)	40.94 (8.11)	42.77 (8.58)	.16	25 (50)	41.55 (8.86)	42.39 (9.47)	.24	.48		.63	
		Mental component	24 (52)	46.42 (12.21)	43.77 (12.28)	.18	25 (50)	46.96 (11.21)	48.31 (10.51)	.53	.73		.37	
	**HADS**													
		Anxiety	24 (52)	6.94 (4.38)	7.33 (5.07)	.47	22 (44)	6 (4)	5.97 (3.5)	.50	.06		.12	
		Depression	23 (50)	5.09 (3.95)	5.51 (4.67)	.73	23 (46)	5.21 (3.85)	5.55 (4.2)	.92	.77		.32	
SEMCD6			26 (57)	7.23 (2.18)	7.35 (1.57)	.73	24 (48)	7.08 (2.55)	6.75 (2.21)	.53	.13		.42	
	**Self-report**													
		Hospital (number of visits)	28 (61)	4.29 (10.56)	1.43 (4.11)	.10	23 (46)	3.7 (6.51)	0.35 (1.11)	.02	.02		.32	
		ED^g^ visits	28 (61)	1.29 (4.65)	0.5 (1.33)	.34	23 (46)	0.071 (1.34)	0.22 (0.57)	.07	.12		.31	
		Clinic visits	20 (44)	3.54 (4.11)	3.71 (2.85)	.52	21 (42)	4.19 (6.01)	2.95 (4.61)	.39	.39		.12	
		Family physician visits	25 (54)	1.65 (1.22)	1.28 (1.62)	.49	23 (46)	2.48 (3.72)	1.85 (2.23)	.27	.28		.07	
	**SCHFI**													
		Maintenance	24 (52)	73.42 (14.77)	79.83 (15.81)	.04	17 (34)	73.64 (15.75)	76.18 (17.48)	.49	.74		.82	
		Management	24 (52)	71.75 (15.64)	75.42 (19.83)	.42	17 (34)	64.47 (27.51)	69.53 (26.53)	.40	.67		.40	
		Confidence	24 (52)	69.43 (15.49)	71.83 (19.74)	.50	16 (32)	69.94 (25.09)	76.38 (19.86)	.16	.92		.34	
	**MLHFQ**													
		Total	23 (50)	40.85 (28.52)	35.88 (23.65)	.12	17 (34)	42.81 (26.15)	31.55 (29.45)	.07	.67		.10	
		Physical	23 (50)	19.13 (11.01)	14.58 (10.9)	.046	17 (34)	20.78 (10.64)	19.05 (12.4)	.11	.43		.06	
		Emotional	22 (48)	10.38 (7.97)	9 (7.45)	.81	15 (30)	11.38 (7.08)	9.33 (7.1)	.10	.64		.20	

^a^A 2-tailed *t* test was used.

^b^SF-36: 36-Item Short Form Health Survey questionnaire.

^c^HADS: Hospital Anxiety and Depression Scale.

^d^SEMCD6: Self-Efficacy for Managing Chronic Disease 6-Item scale.

^e^MLHFQ: Minnesota Living with Heart Failure Questionnaire.

^f^SCHFI: Self-Care of Heart Failure Index.

^g^ED: emergency department.

Self-care maintenance and physical quality of life improved significantly for patients with HF in the telemonitoring group (*P*=.04 and *P*=.046, respectively). However, none of the between-group comparisons were statistically significant, likely because of the general improvement in self-care and quality of life scores of the control group and insufficient sample size for detecting changes in condition-specific metrics.

### Telemonitoring Use

**Figure 3:**
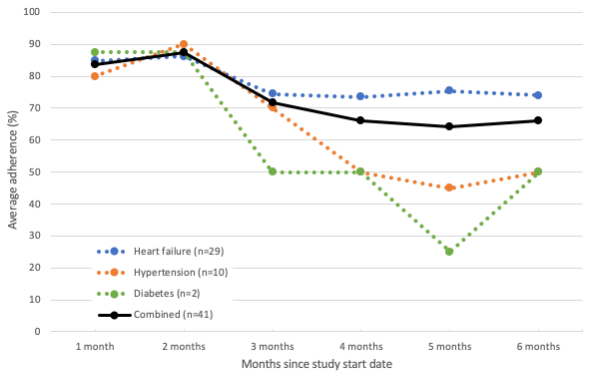
Average combined and condition-specific adherence rates over time.

Of the 41 patients who completed the study in the intervention arm, 29 (71%) used the HF module, 10 (24%) used the HT module, and 2 (5%) used the DM module. On average, patients with HF completed all 4 readings on 78.1% (SD 5.8%) of the days that they were enrolled in the 6-month trial. This use rate increased to 86.2% when looking at the percentage of days the patients with HF reported at least one reading. Average adherence was 64.2% (SD 18.6%) for the patients with HT and 58.3% (SD 24.6%) for the patients with DM based on a minimum of biweekly and weekly readings for HT and DM, respectively. The combined average adherence across all participants was 73.1% (SD 10%) over the study period. Figure 3 depicts the monthly adherence rates for the 3 disease modules. It shows that although adherence was initially high across all conditions and remained relatively stable for patients with HF, it dropped markedly after the second month for patients with HT and DM.

### Experiences With the Telemonitoring Program

#### Overview

Of the 41 patients who completed the study in the intervention arm, 13 (32%) were interviewed, including 62% (8/13) of patients with HF, 31% (4/13) of patients with HT, and 8% (1/13) of patients with DM. The HCPs included 1 cardiologist, 2 cardiac nurse practitioners, 1 HT specialist, and 1 endocrinologist. The interview findings, which contributed to explaining the overall null results of the study, are summarized in the following themes: (1) challenges of implementing MCC telemonitoring in a siloed health care system, (2) perceptions of the telemonitoring system, (3) perceived benefits differed based on the condition monitored, and (4) opportunities for MCC telemonitoring.

#### Theme 1: Challenges of Implementing MCC Telemonitoring in a Siloed Health Care System

Patients and HCPs expressed that the telemonitoring intervention did not adequately address the challenge of delivering and coordinating advanced care for patients with MCCs. Although this was due in part to development delays that resulted in patients with different conditions undergoing the study at different times, there were also no formal communication processes in place among relevant specialties that would have enabled optimal multidisciplinary care:

How do you integrate information from multiple specialists...I don’t have multiple chronic conditions, I have one condition...I didn’t do the blood sugar stuff, I didn’t do the heart failure stuff, I didn’t do the chronic obstructive pulmonary disease stuff, I didn’t do the mental health stuff...The people that would handle all these conditions simultaneously would be family doctors, except family doctors often don’t have the expertise to handle complex conditions.

HCP 5

As the model of care did not connect existing specialty siloes, the clinical processes surrounding the use of the telemonitoring system were different based on the recruitment setting. For HF, an HCP described that a strong, team-based model with clearly defined clinical roles enabled rapid clinical response to worsening conditions:

It’s given me different work to do...although [I’m] not complaining about that because that is part of why I’m hired. It’s just that...in order for [a telemonitoring program] to work, you have to have a clinician who is devoting time to do all of that, to answer alerts, to document, and to see patients that are unwell in clinic or fax them lab work [requisitions].

HCP 3

In contrast, DM and HT alerts were directly sent to the physicians who reflected on the absence of an appropriate model of care to support telemonitoring within those clinics and how it might have been better if the information was sent to a different member of the team:

The difference between the heart failure and the hypertension is that the heart failure has a whole system in place. We don’t have a similar type of system for the hypertension, I assume for the diabetes as well...the physician is the last person that needs the information. It’s their nurse and dietician.

HCP 4

#### Theme 2: Perceptions of the Telemonitoring System

Most patients had positive opinions about the telemonitoring system and found it easy to take readings and navigate the different features:

It’s pretty easy to use...I loved the fact that you took your sugar and it connected right away to the phone...I think that for my purposes, it did everything I needed it to do. You got some averages in there too which I liked, you could see [readings] over a period of time.

Patient with DM 1

Similarly, patients largely appreciated the automated self-care messages, with the exception of a minority who expressed confusion about messages directing them to contact the clinic when a reading was out of their target range. Some patients, across all conditions, recounted not knowing where to call and what to say when they did. In addition, some patients with HF reported ignoring such messages as they realized that their care team would contact them in the case of a true concern:

I think that perhaps the instructions in the system of the Medly program were maybe unclear. Sometimes there would be a prompt to call...[but] then I would call and there wouldn’t really be a sort of a good answer on why I should be calling.

Patient with HT 3

It’ll say “contact the heart clinic or go to your hospital if you don’t feel well”. I’ve just gotten so used to it. Say my weight is up two pounds or something, I know it’s not critical...it says call, but I haven’t been...I know that [my care team] will call me and make sure everything’s okay.

Patient with HF 26

Unlike patients, there were mixed opinions about the telemonitoring system among HCPs. These differences were attributed to the physiological nature of the chronic condition and the existing standard of care in which the intervention was implemented. For example, owing to the dynamic nature of HF and the potential for rapid decompensation that could lead to hospitalization, telemonitoring alerts typically require immediate action from patients or HCPs. Therefore, HF HCPs were motivated to find ways of incorporating the viewing of telemonitoring data and alerts within their existing workflows:

I think the issue for clinicians is that they’re building this into their day-to-day busy practice so there are things like that I think can be done [to improve the system] but generally I think the dashboard is pretty sophisticated, it’s easy to use, [and] incredibly easy to navigate.

HCP 2

Conversely, DM and HT HCPs expressed feeling that the system was built on the premise of identifying an acute change (needed for HF) rather than communicating information about longer-term trends. As the system was not perceived to convey DM and HT telemonitoring data to physicians in a way that is consistent with the existing management of those conditions, there was less incentive to incorporate the use of the system within their workflows:

I didn’t get any information from [the system]...I got no monthly reports about any of the individuals. The only thing I got was an occasional alert if their blood pressure was extraordinarily high...What you want to do is to map the progress of the patient since their blood pressure is not controlled. Even if it’s not an alert, think if you had high blood pressure and your blood pressure was not in the alert level, but it was not controlled. Do you want your provider not to have the information?

HCP 5

Finally, a factor specific to DM was the fact that the data from the newer, and increasingly prominent, flash glucose monitors could not be sent to the Medly telemonitoring system:

To be honest with you, I kind of stopped using it halfway through because...when I mentioned I was doing the study at the last appointment, [my doctor] said, “Well I guess it’s irrelevant, based on the new technologies that are out there for checking your glucose.”...he made it sound like that the study wasn’t happening anymore.

Patient with DM 1

#### Theme 3: Perceived Benefits Differed Based on the Condition Monitored

Opinions of the models of care and telemonitoring system previously described contributed to the perceived benefits of the intervention, and importantly, how these differed for patients with HF, DM, and HT and HCPs. Patients who were followed for HF expressed that the intervention enabled self-management by helping them form a routine of taking daily readings and associating those results with their behavior. The frequency of data collection and urgency-based alerts sent to the clinical team also contributed to the perception of improved clinical management:

Every morning when I get up, that’s the first thing I do and it kind of gives me an overview of my condition...I wake up and I know I’ve been having problems breathing and when I check my weight, I say, okay, I have gained weight...three pounds. I say okay and tie [it] back to what I’ve eaten the night before.

Patient with HF 8

The close monitoring offered by the HF telemonitoring system and model of care led to patients with HF benefiting from peace of mind and a closer relationship with their care team:

It gives you peace of mind, because...the doctor’s seeing it, which is important, because...when the people who are really interested in your well-being are that far away, it’s nice that you’ve got that system in place, where if something goes wrong you know right away.

Patient with HF 2

Patients followed up for HT and DM expressed many of the same self-management benefits as the patients with HF, including the idea that the system encouraged them to take blood pressure and blood glucose readings more regularly than they normally would and contributed to a greater awareness of life factors that could influence those readings:

I discovered...triggers of my problem in blood pressure. Mainly, for example, when I have a little constipation, it has an effect on high blood pressure. Or, I had, for example, a little stress that affected me...Medly allowed me to be more aware of the situation. It’s a mindfulness program. I became aware of what I was doing.

Patient with HT 7

However, unlike patients with HF, patients with HT and DM did not express the same peace of mind and closer therapeutic relationship with their HCPs as a result of telemonitoring. This was explained by the lack of feedback from HCPs and the perception that HCPs were not reviewing the data to the degree that the patients had expected:

I didn’t really have any sort of feedback from a physician’s point of view as to what the Medly information was telling them. That would have been interesting...[and] made the whole experience sort of more sensible. Otherwise, I’m just slapping a cuff on my arm and taking a reading and so if it was more interactive, it might have had more meaning.

Patient with HT 3

This perception was confirmed by the HT and DM HCPs, who felt that the intervention did not affect their clinical management of patients because of the fact that the system did not provide health data in a meaningful way:

I had virtually no interaction with the study...I wouldn’t have even known if patients came for follow-up, whether they were in the study or not. They didn’t volunteer the information; I didn’t give any information from MCC about the patients. There was no feedback [from the system].

HCP 5

#### Theme 4: Opportunities for MCC Telemonitoring

Despite the challenges with this trial, HCPs spoke of the potential of telemonitoring, if designed appropriately, to help address the existing limitations of virtual care that is becoming the norm since the onset of the COVID-19 pandemic:

You need data points, you can’t [provide virtual care] in a vacuum and Medly is a system whereby you can get the data points...What telemonitoring is supposed to do is to give a person a profile between clinic visits and if I can't get that through the regular virtual service, this is where the telemonitoring services come in. [However], it's got to be structured, it's got to be done in a systematic way, and it's got to be done so that the patients can submit the information. The benefit for the patient is then they get feedback between clinic visits, or if they don't get feedback between clinic visits, at least the physician at the clinic visits has reliable information in order to ascertain what next steps have to be done to improve management.

HCP 5

In addition to the need to present data in a meaningful way, as highlighted in theme 2, there was consensus among HCPs that a telemonitoring system, especially one for MCC, needs to be integrated with the existing organizational information systems:

It has to be integrated with a larger system-wide electronic solution...It duplicates work when I have to open a chart, and open a computer program, and transcribe things manually into the chart...Getting alerted is good but [the] communication piece and that documentation piece is [critical].

HCP 4

In assessing the value of telemonitoring, HCPs did not disassociate the design of the telemonitoring system from the design of the model of care. For telemonitoring of complex conditions to be feasible, they envisioned a team-based approach in which a nonphysician (eg, nurse, nurse practitioner, or allied health professional) held a central role in the intervention because of a greater alignment of the scope of practice and existing remuneration models:

I guess for nurses and dieticians who are employed by a hospital, [the issue of remuneration] is irrelevant because they have a salary, so it could easily be integrated into their current workflow. But for physicians their only real commodity is their time...so if this is going to add to the current work, or displace current remunerated work it's a no-go.

HCP 4

You could have a nurse practitioner who leads these programs and works with a staff nurse or that kind of helps triage and call patients back and manage that way but then also a physician if there’s a really big problem that you have to go to...you could look at different models of care for these programs.

HCP 2

## Discussion

### Principal Findings

In the absence of high-quality evidence on telemonitoring for managing MCCs [48], this study sought to evaluate the impact of a mobile phone–based telemonitoring program to manage patients with complex conditions in specialty care settings. Through a pragmatic RCT, halted before reaching the intended sample size, we observed no effects of the telemonitoring program on health status, anxiety and depression, self-efficacy, and most health service use metrics. Subgroup analyses of patients with HF in the intervention arm found a significant improvement in physical quality of life and self-care maintenance; however, no differences in these groups were observed between the treatment arms. The reduction in self-reported hospitalization found in the control group could also be seen as a trend in the intervention group but did not reach statistical significance, likely because of the small sample size. This reduction in both arms may be partly attributed to the impact of the HF clinic in stabilizing patients.

Several challenges were encountered during the trial, necessitating deviations from the published protocol, which likely contributed to the null results of the study. Foremost, this trial did not reach its intended sample size of 146. Although the onset of the COVID-19 pandemic and the rapid shift to virtual care for both arms did bring about the decision to stop recruitment permanently, other important challenges contributed to slow recruitment. Importantly, funding and development challenges contributed to the inability to include the planned chronic obstructive pulmonary disease and chronic kidney disease modules. Similarly, although patients who participated in our study had MCCs, we were unable to offer monitoring for all the conditions simultaneously as the HF, HT, and DM modules were not initially available at the same time. Therefore, the MCC model of care was never fully tested as there was never a need to explore communication workflows for multidisciplinary care that are inherent to the care of MCCs. Finally, recruitment of patients from the diabetes clinic was challenging because of the emergence of newer continuous glucose monitoring technologies at the time of recruitment.

Although patient perceptions of the telemonitoring program were largely positive, the perceived benefits and, consequently, use of the system varied across conditions. Indeed, although all patients in this study started with high adherence, these higher levels were only maintained in patients who were followed for HF. These differences may reflect the more actionable nature of the self-care instructions and more frequent clinician alerts from the HF module relative to those provided by the HT and DM modules. HF tends to be more dynamic and requires more parameters to be taken at a higher frequency, which provides the data inputs necessary for a more sophisticated telemonitoring algorithm. Conversely, HT and DM require fewer inputs at a lower frequency, which typically means that less urgency is required when alerts are generated. This does not suggest that HT and DM are not suitable conditions for systems such as Medly, as patients followed up for these conditions did express benefiting from self-management support. Rather, the opinions expressed by the HT and DM HCPs suggest that the way telemonitoring data are presented to HCPs needs to consider the greater importance of long-term trends versus the acute symptom-based alerting needed for optimal HF management. Considering these differences between conditions may increase the perceived relative advantage [46] of telemonitoring to promote comparable use across conditions.

Various factors related to the implementation of the telemonitoring program may also explain the lack of impact observed. We posit that an initially narrow conceptualization of telemonitoring, focused primarily on the technology rather than the model of care, may have contributed to these implementation issues. For instance, relative to HF [49], minimal consideration was given to the model of care associated with the DM and HT modules. Consistent with previous studies, we recommend that future research conceive telemonitoring as a *program* comprising both the *system* and its associated *model of care*. Critically, implementation study designs should be used to assess the feasibility of the program before conducting a trial so that challenges may be identified and addressed [50].

A notable characteristic thought to enhance the feasibility of telemonitoring for patients with complex conditions and MCCs was having a nurse in a central role as it relates to alert management and care coordination. In contrast to the fee-for-service model for physicians, all HCPs in this study noted that the salaried remuneration of nurses offered greater flexibility needed to monitor patients with MCCs through telemonitoring. Indeed**,** when the same MCC telemonitoring system was applied in a different study within a multidisciplinary nurse-led model of care [51], telemonitoring was not only perceived positively by patients with MCCs but was also normalized within their daily life and routines [52], which was a finding that was only observed among the patients with HF and HCPs in this trial, who also benefited from a nurse-led model. What may explain the stark contrast in the results between these studies is the differences in their underlying program theory. Per Kastner et al [53], effective interventions for older adults with MCCs are often founded upon principles of care coordination, disease prioritization, and patient self-management. From this, we conclude that telemonitoring interventions for patients with complex conditions, especially those with MCCs, should (1) be integrated with usual care workflows and complementary clinical services, (2) be supported by a multidisciplinary model, and (3) include ≥1 element of care coordination (eg, referral pathways, case management, and interoperable information systems). Critically, these 3 elements may be best operationalized by having a nurse play a central role in the intervention.

### Comparison With Previous Work

The outcomes of this trial are consistent with recent reviews [48,54], indicating a dearth of evidence on the effectiveness of telemedicine interventions for the population with MCC. In 2020, Kraef et al [48] identified only 1 study reporting no impact of telemedicine on self-reported health status and only moderate improvements in disease control measures such as hemoglobin A_1c_ and systolic blood pressure across 7 meta-analyzed studies. Similarly, Pisa et al [54] found no impact of telemedicine interventions in primary care on quality of life and contradictory results on ED visits and hospital admissions across 5 studies [54].

Importantly, trials reporting significant effects on condition-specific outcomes and no implementation challenges often included a role of a nonphysician HCP in the intervention, most commonly a nurse [30,55-57]. Moreover, several authors have attributed frequent and immediate feedback to patient data as important facilitators in promoting the intended use of telemonitoring systems and the success of their trials [30,57,58]. Thus, the results of this trial and the existing literature converge in identifying care coordination as an important component in telemonitoring interventions for MCCs. We suggest that this be considered as a central component of further studies exploring the feasibility and effectiveness of telemonitoring for MCCs.

### Limitations

In addition to the recruitment and implementation challenges previously discussed, this study had limitations attributable to the trial’s pragmatic nature. First, although the study period was 6 months, the study duration lasted approximately 4 years with no overlap in the periods in which patients with HF and patients with HT or DM were active. This meant that in addition to inhibiting the exploration of multidisciplinary care coordination, ending the recruitment because of the COVID-19 pandemic contributed to a difference in sample size across conditions. Second, self-reported health service use introduces potential challenges because of recall. Third, unintended selection bias is possible as the recruitment relied on busy clinicians identifying eligible patients during clinic hours. We do not have data on patients who might have been eligible but were never identified by their care team or on patients who were approached but did not agree to be introduced to the research coordinator. Finally, the interviews were conducted with a convenience sample of patients, and although all HCPs who were involved in the study in any significant manner were interviewed, the number of 5 HCPs was relatively small.

### Conclusions

Decades of research suggest that telemonitoring for self-management and clinical decision support may assist in managing single chronic conditions; however, its effectiveness for the growing number of patients with complex conditions, including those with MCCs, remains unclear. This 6-month pragmatic RCT sought to evaluate the impact on patients and HCPs and their experiences with a telemonitoring program to manage patients with complex conditions compared with those in standard care. No significant within- and between-group differences were found in the primary outcome of health status or the secondary outcomes of self-efficacy, anxiety, depression, and health service use. However, improvements in physical quality of life and self-care maintenance were observed in patients with HF who were enrolled in the telemonitoring program. Qualitative data suggest that these null findings may be because of the fact that not all disease modules were available at the same time, implementation challenges within the siloed specialty care, varying degrees of acuity and urgency across chronic conditions, and an insufficient sample size attributable to the onset of the COVID-19 pandemic. On the basis of the study findings, we caution that self-management and clinical decision support are necessary but not sufficient components of telemonitoring for patients with complex conditions. We conclude that telemonitoring for patients with complex conditions or those within multidisciplinary care settings may be best operationalized through nurse-led models of care and that the complex nature of these interventions makes it such that they are best studied through feasibility or pragmatic study designs before conducting RCTs.

## Appendix

Multimedia Appendix 1 CONSORT-eHEALTH Checklist (V 1.6.1).

## Abbreviations

DM: diabetes mellitus
ED: emergency department
HCP: health care provider
HF: heart failure
HT: hypertension
MCC: multiple chronic condition
RCT: randomized controlled trial
UHN: University Health Network
