# Supplementary material for: Challenges of Telemonitoring Programs for Complex Chronic Conditions: Randomized Controlled Trial With an Embedded Qualitative Study
Source: J Med Internet Res. 2022 Jan 26;24(1):e31754. doi: 10.2196/31754 (PMC8829695; doi:10.2196/31754)
Supplement: Multimedia Appendix 1 [file jmir_v24i1e31754_app1.pdf]

CONSORT-EHEALTH (V 1.6.1) - Submission/Publication Form

The CONSORT-EHEALTH checklist is intended for authors of randomized trials evaluating web-based and Internet-based applications/interventions, including mobile interventions, electronic games (incl multiplayer games), social media, certain telehealth applications, and other interactive and/or networked electronic applications. Some of the items (e.g. all subitems under item 5 - description of the intervention) may also be applicable for other study designs.

The goal of the CONSORT EHEALTH checklist and guideline is to be
a) a guide for reporting for authors of RCTs,
b) to form a basis for appraisal of an ehealth trial (in terms of validity)

CONSORT-EHEALTH items/subitems are MANDATORY reporting items for studies published in the Journal of Medical Internet Research and other journals / scientific societies endorsing the checklist.

Items numbered 1., 2., 3., 4a., 4b etc are original CONSORT or CONSORT-NPT (non-pharmacologic treatment) items.
Items with Roman numerals (i., ii, iii, iv etc.) are CONSORT-EHEALTH extensions/clarifications.

As the CONSORT-EHEALTH checklist is still considered in a formative stage, we would ask that you also RATE ON A SCALE OF 1-5 how important/useful you feel each item is FOR THE PURPOSE OF THE CHECKLIST and reporting guideline (optional).

Mandatory reporting items are marked with a red *.
In the textboxes, either copy & paste the relevant sections from your manuscript into this form - please include any quotes from your manuscript in QUOTATION MARKS,
or answer directly by providing additional information not in the manuscript, or elaborating on why the item was not relevant for this study.

YOUR ANSWERS WILL BE PUBLISHED AS A SUPPLEMENTARY FILE TO YOUR PUBLICATION IN JMIR AND ARE CONSIDERED PART OF YOUR PUBLICATION (IF ACCEPTED).
Please fill in these questions diligently. Information will not be copyedited, so please use proper spelling and grammar, use correct capitalization, and avoid abbreviations.

DO NOT FORGET TO SAVE AS PDF _AND_ CLICK THE SUBMIT BUTTON SO YOUR ANSWERS ARE IN OUR DATABASE !!!

Citation Suggestion (if you append the pdf as Appendix we suggest to cite this paper in the caption):
Eysenbach G, CONSORT-EHEALTH Group
CONSORT-EHEALTH: Improving and Standardizing Evaluation Reports of Web-based and

Mobile Health Interventions
J Med Internet Res 2011;13(4):e126
URL: http://www.jmir.org/2011/4/e126/
doi: 10.2196/jmir.1923
PMID: 22209829

* Required

Your name *

First Last

Amika Shah

Primary Affiliation (short), City, Country *

University of Toronto, Toronto, Canada

University of Toronto, Toronto, Canada

Your e-mail address *

abc@gmail.com

amika.shah@mail.utoronto.ca

Title of your manuscript *

Provide the (draft) title of your manuscript.

Challenges of Telemonitoring Programs for Complex Chronic Conditions: A Randomized Controlled Trial with an Embedded Qualitative Study

Name of your App/Software/Intervention *

If there is a short and a long/alternate name, write the short name first and add the long name in brackets.

Medly

Evaluated Version (if any)

e.g. "V1", "Release 2017-03-01", "Version 2.0.27913"

Your answer

Language(s) *

What language is the intervention/app in? If multiple languages are available, separate by comma (e.g. "English, French")

English

URL of your Intervention Website or App

e.g. a direct link to the mobile app on app in appstore (itunes, Google Play), or URL of the website. If the intervention is a DVD or hardware, you can also link to an Amazon page.

https://play.google.com/store/apps/details?id=org.ehealthinnovation.medly ; https://apps.ap

URL of an image/screenshot (optional)

Your answer

Accessibility *

Can an enduser access the intervention presently?

- ○ access is free and open
- ◉ access only for special usergroups, not open
- ○ access is open to everyone, but requires payment/subscription/in-app purchases
- ○ app/intervention no longer accessible
- ○ Other:

Primary Medical Indication/Disease/Condition *

e.g. "Stress", "Diabetes", or define the target group in brackets after the condition, e.g. "Autism (Parents of children with)", "Alzheimers (Informal Caregivers of)"

Heart failure; hypertension; diabetes

Primary Outcomes measured in trial *

comma-separated list of primary outcomes reported in the trial

Health status

Secondary/other outcomes

Are there any other outcomes the intervention is expected to affect?

Anxiety, depression, self-efficacy to manage chronic condition, number of self-reported interactions with the health system, self-care, heart failure-specific quality of life

Recommended "Dose" *

What do the instructions for users say on how often the app should be used?

- ○ Approximately Daily
- ○ Approximately Weekly
- ○ Approximately Monthly
- ○ Approximately Yearly
- ○ "as needed"
- ● Other: Heart failure (daily); hypertension (once every two weeks); diabetes (a

Approx. Percentage of Users (starters) still using the app as recommended after 3 months *

- ○ unknown / not evaluated
- ○ 0-10%
- ○ 11-20%
- ○ 21-30%
- ○ 31-40%
- ○ 41-50%
- ○ 51-60%
- ○ 61-70%
- ● 71%-80%
- ○ 81-90%
- ○ 91-100%
- ○ Other:

Overall, was the app/intervention effective? *

- ( ) yes: all primary outcomes were significantly better in intervention group vs control
- ( ) partly: SOME primary outcomes were significantly better in intervention group vs control
- (x) no statistically significant difference between control and intervention
- ( ) potentially harmful: control was significantly better than intervention in one or more outcomes
- ( ) inconclusive: more research is needed
- ( ) Other:

Article Preparation Status/Stage *

At which stage in your article preparation are you currently (at the time you fill in this form)

- ( ) not submitted yet - in early draft status
- ( ) not submitted yet - in late draft status, just before submission
- (x) submitted to a journal but not reviewed yet
- ( ) submitted to a journal and after receiving initial reviewer comments
- ( ) submitted to a journal and accepted, but not published yet
- ( ) published
- ( ) Other:

Journal *

If you already know where you will submit this paper (or if it is already submitted), please provide the journal name (if it is not JMIR, provide the journal name under "other")

- ○ not submitted yet / unclear where I will submit this
- ◉ Journal of Medical Internet Research (JMIR)
- ○ JMIR mHealth and UHealth
- ○ JMIR Serious Games
- ○ JMIR Mental Health
- ○ JMIR Public Health
- ○ JMIR Formative Research
- ○ Other JMIR sister journal
- ○ Other:

Is this a full powered effectiveness trial or a pilot/feasibility trial? *

- ◉ Pilot/feasibility
- ○ Fully powered

Manuscript tracking number *

If this is a JMIR submission, please provide the manuscript tracking number under "other" (The ms tracking number can be found in the submission acknowledgement email, or when you login as author in JMIR. If the paper is already published in JMIR, then the ms tracking number is the four-digit number at the end of the DOI, to be found at the bottom of each published article in JMIR)

- ○ no ms number (yet) / not (yet) submitted to / published in JMIR
- ◉ Other: 31754

TITLE AND ABSTRACT

1a) TITLE: Identification as a randomized trial in the title

1a) Does your paper address CONSORT item 1a? *

I.e does the title contain the phrase "Randomized Controlled Trial"? (if not, explain the reason under "other")

- (selected) yes
- Other:

1a-i) Identify the mode of delivery in the title

Identify the mode of delivery. Preferably use "web-based" and/or "mobile" and/or "electronic game" in the title. Avoid ambiguous terms like "online", "virtual", "interactive". Use "Internet-based" only if Intervention includes non-web-based Internet components (e.g. email), use "computer-based" or "electronic" only if offline products are used. Use "virtual" only in the context of "virtual reality" (3-D worlds). Use "online" only in the context of "online support groups". Complement or substitute product names with broader terms for the class of products (such as "mobile" or "smart phone" instead of "iphone"), especially if the application runs on different platforms.

	1	2	3	4	5	
subitem not at all important	○	○	○	○	○	essential

Does your paper address subitem 1a-i? *

Copy and paste relevant sections from manuscript title (include quotes in quotation marks "like this" to indicate direct quotes from your manuscript), or elaborate on this item by providing additional information not in the ms, or briefly explain why the item is not applicable/relevant for your study

"Challenges of Telemonitoring Programs for Complex Chronic Conditions: A Randomized Controlled Trial with an Embedded Qualitative Study"

1a-ii) Non-web-based components or important co-interventions in title

Mention non-web-based components or important co-interventions in title, if any (e.g., “with telephone support”).

	1	2	3	4	5	
subitem not at all important	○	○	○	○	○	essential

Does your paper address subitem 1a-ii?

Copy and paste relevant sections from manuscript title (include quotes in quotation marks "like this" to indicate direct quotes from your manuscript), or elaborate on this item by providing additional information not in the ms, or briefly explain why the item is not applicable/relevant for your study

"Challenges of Telemonitoring Programs for Complex Chronic Conditions: A Randomized Controlled Trial with an Embedded Qualitative Study"

The term program was intentionally used in the manuscript title to encompass the clinical decision support and self-care components of telemonitoring interventions.

1a-iii) Primary condition or target group in the title

Mention primary condition or target group in the title, if any (e.g., “for children with Type I Diabetes”)
Example: A Web-based and Mobile Intervention with Telephone Support for Children with Type I Diabetes: Randomized Controlled Trial

	1	2	3	4	5	
subitem not at all important	○	○	○	○	○	essential

Does your paper address subitem 1a-iii? *

Copy and paste relevant sections from manuscript title (include quotes in quotation marks "like this" to indicate direct quotes from your manuscript), or elaborate on this item by providing additional information not in the ms, or briefly explain why the item is not applicable/relevant for your study

"Challenges of Telemonitoring Programs for Complex Chronic Conditions: A Randomized Controlled Trial with an Embedded Qualitative Study"

1b) ABSTRACT: Structured summary of trial design, methods, results, and conclusions

NPT extension: Description of experimental treatment, comparator, care providers, centers, and blinding status.

1b-i) Key features/functionalities/components of the intervention and comparator in the METHODS section of the ABSTRACT

Mention key features/functionalities/components of the intervention and comparator in the abstract. If possible, also mention theories and principles used for designing the site. Keep in mind the needs of systematic reviewers and indexers by including important synonyms. (Note: Only report in the abstract what the main paper is reporting. If this information is missing from the main body of text, consider adding it)

	1	2	3	4	5	
subitem not at all important	○	○	○	○	○	essential

Does your paper address subitem 1b-i? *

Copy and paste relevant sections from the manuscript abstract (include quotes in quotation marks "like this" to indicate direct quotes from your manuscript), or elaborate on this item by providing additional information not in the ms, or briefly explain why the item is not applicable/relevant for your study

"Participants were randomized into the control and telemonitoring groups with the latter being instructed to take readings relevant to their condition(s). The telemonitoring system contained an algorithm that generated decision support in the form of actionable self-care directives to patients and alerts to HCPs."

1b-ii) Level of human involvement in the METHODS section of the ABSTRACT

Clarify the level of human involvement in the abstract, e.g., use phrases like “fully automated” vs. “therapist/nurse/care provider/physician-assisted” (mention number and expertise of providers involved, if any). (Note: Only report in the abstract what the main paper is reporting. If this information is missing from the main body of text, consider adding it)

	1	2	3	4	5	
subitem not at all important	○	○	○	○	○	essential

Does your paper address subitem 1b-ii?

Copy and paste relevant sections from the manuscript abstract (include quotes in quotation marks "like this" to indicate direct quotes from your manuscript), or elaborate on this item by providing additional information not in the ms, or briefly explain why the item is not applicable/relevant for your study

"The telemonitoring system contained an algorithm that generated decision support in the form of actionable self-care directives to patients and alerts to HCPs."

1b-iii) Open vs. closed, web-based (self-assessment) vs. face-to-face assessments in the METHODS section of the ABSTRACT

Mention how participants were recruited (online vs. offline), e.g., from an open access website or from a clinic or a closed online user group (closed usergroup trial), and clarify if this was a purely web-based trial, or there were face-to-face components (as part of the intervention or for assessment). Clearly say if outcomes were self-assessed through questionnaires (as common in web-based trials). Note: In traditional offline trials, an open trial (open-label trial) is a type of clinical trial in which both the researchers and participants know which treatment is being administered. To avoid confusion, use “blinded” or “unblinded” to indicated the level of blinding instead of “open”, as “open” in web-based trials usually refers to “open access” (i.e. participants can self-enrol). (Note: Only report in the abstract what the main paper is reporting. If this information is missing from the main body of text, consider adding it)

	1	2	3	4	5	
subitem not at all important	○	○	○	○	○	essential

Does your paper address subitem 1b-iii?

Copy and paste relevant sections from the manuscript abstract (include quotes in quotation marks "like this" to indicate direct quotes from your manuscript), or elaborate on this item by providing additional information not in the ms, or briefly explain why the item is not applicable/relevant for your study

"A pragmatic, unblinded, 6-month RCT sought to recruit 146 patients with a diagnosis of heart failure (HF), uncontrolled hypertension (HT), and/or insulin requiring diabetes (DM) from outpatient specialty settings in Toronto, Canada."

1b-iv) RESULTS section in abstract must contain use data

Report number of participants enrolled/assessed in each group, the use/uptake of the intervention (e.g., attrition/adherence metrics, use over time, number of logins etc.), in addition to primary/secondary outcomes. (Note: Only report in the abstract what the main paper is reporting. If this information is missing from the main body of text, consider adding it)

	1	2	3	4	5	
subitem not at all important	○	○	○	○	○	essential

Does your paper address subitem 1b-iv?

Copy and paste relevant sections from the manuscript abstract (include quotes in quotation marks "like this" to indicate direct quotes from your manuscript), or elaborate on this item by providing additional information not in the ms, or briefly explain why the item is not applicable/relevant for your study

"The primary outcome was health status as measured by the SF-36. Secondary outcomes included anxiety and depression, self-efficacy in chronic disease management, and self-reported healthcare utilization. HF-related quality of life and self-care measures were also collected from patients followed for HF."

"A total of 96 patients were recruited and randomized. Recruitment was terminated early due to implementation challenges and the onset of COVID-19."

1b-v) CONCLUSIONS/DISCUSSION in abstract for negative trials

Conclusions/Discussions in abstract for negative trials: Discuss the primary outcome - if the trial is negative (primary outcome not changed), and the intervention was not used, discuss whether negative results are attributable to lack of uptake and discuss reasons. (Note: Only report in the abstract what the main paper is reporting. If this information is missing from the main body of text, consider adding it)

	1	2	3	4	5	
subitem not at all important	○	○	○	○	○	essential

Does your paper address subitem 1b-v?

Copy and paste relevant sections from the manuscript abstract (include quotes in quotation marks "like this" to indicate direct quotes from your manuscript), or elaborate on this item by providing additional information not in the ms, or briefly explain why the item is not applicable/relevant for your study

"Opinions expressed by the 5 HCPs and 13 patients interviewed differed based on the condition(s) monitored. Although HF patients reported benefitting from actionable self-care guidance and meaningful interactions with their HCPs, patient and HCP users of the DM and HT modules did not think telemonitoring improved the clinical management of those conditions to the same degree. These differing experiences were largely attributed to the siloed nature of specialty care, and the design of the decision support whereby it was indicated that fluctuations in HT and DM patient status typically required less urgent intervention compared to HF."

"Consistent with previous studies, we recommend that future research conceive telemonitoring as a program and that self-management and clinical decision support are necessary, but not sufficient components of such programs for complex patients with lower acuity. We conclude that a multidisciplinary model of care that includes care coordination must accompany telemonitoring systems which may best be operationalized through novel models of care, such as nurse-led models."

INTRODUCTION

2a) In INTRODUCTION: Scientific background and explanation of rationale

2a-i) Problem and the type of system/solution

Describe the problem and the type of system/solution that is object of the study: intended as stand-alone intervention vs. incorporated in broader health care program? Intended for a particular patient population? Goals of the intervention, e.g., being more cost-effective to other interventions, replace or complement other solutions? (Note: Details about the intervention are provided in “Methods” under 5)

	1	2	3	4	5	
subitem not at all important	○	○	○	○	○	essential

Does your paper address subitem 2a-i? *

Copy and paste relevant sections from the manuscript (include quotes in quotation marks "like this" to indicate direct quotes from your manuscript), or elaborate on this item by providing additional information not in the ms, or briefly explain why the item is not applicable/relevant for your study

"Despite a growing prevalence of patients with complex and multiple chronic conditions (MCCs) [1,2], siloed care models focused on single conditions have been a barrier to the appropriate management of these patients [3]. In Canada, 12.9% of individuals across all age groups report having two or more chronic conditions, and 3.9% report having three or more [1]. These are among the highest cost users of healthcare systems due to a higher frequency of hospitalizations, many of which are thought to be preventable [4,5]. Research suggests that effective patient self-management can reduce the need for urgent care while promoting self-efficacy, improving quality of life, and reducing the risk of adverse psychological effects [6]. However, complex decision making and often conflicting clinical advice from multiple siloed healthcare providers (HCPs) make self-management challenging for patients with MCCs [7,8].

Telemonitoring has the potential to empower patients and HCPs by facilitating patient self-management and clinical decision support to manage MCCs. Telemonitoring systems enable patients to track vital signs and symptoms and can enable automatic generation of self-management instructions [9]. In addition, by delivering these data to the clinical team, HCPs can identify patients showing early signs of exacerbation which offers an opportunity to reinforce principles of self-management at a “teachable moment” [10]. Importantly, telemonitoring allows HCPs to provide remote guidance or make changes to a care plan, thereby stabilizing symptoms before they escalate to a point of hospitalization

2a-ii) Scientific background, rationale: What is known about the (type of) system

Scientific background, rationale: What is known about the (type of) system that is the object of the study (be sure to discuss the use of similar systems for other conditions/diagnoses, if appropiate), motivation for the study, i.e. what are the reasons for and what is the context for this specific study, from which stakeholder viewpoint is the study performed, potential impact of findings [2]. Briefly justify the choice of the comparator.

	1	2	3	4	5	
subitem not at all important	○	○	○	○	○	essential

Does your paper address subitem 2a-ii? *

Copy and paste relevant sections from the manuscript (include quotes in quotation marks "like this" to indicate direct quotes from your manuscript), or elaborate on this item by providing additional information not in the ms, or briefly explain why the item is not applicable/relevant for your study

"Although research on telemonitoring is rapidly growing [11], these systems typically target a singular condition such as diabetes mellitus (DM), hypertension (HT), or heart failure (HF) [12–14]. Systematic reviews indicate that telemonitoring for single conditions lead to improved health outcomes and quality of life, as well as reductions in healthcare utilization and costs [15–21]. Studies that do not report improvements often did not include a self-care component, were difficult to use, or did not target the most ill and frequently hospitalized patients [22–25]. To date, few studies have focused on the use of telemonitoring among complex patients, even though these patients may benefit the most from such interventions [15,26–32]."

2b) In INTRODUCTION: Specific objectives or hypotheses

Does your paper address CONSORT subitem 2b? *

Copy and paste relevant sections from the manuscript (include quotes in quotation marks "like this" to indicate direct quotes from your manuscript), or elaborate on this item by providing additional information not in the ms, or briefly explain why the item is not applicable/relevant for your study

"The objective of this study was to evaluate the impact of a mobile phone-based telemonitoring program for the management of complex patients in specialty care settings. Complex patients were defined as those who are at high risk for hospitalization, exacerbations of their chronic conditions, and disease progression, as well as patients with MCCs, including HF, uncontrolled HT, and insulin-requiring DM. The primary research question was: What is the impact of a telemonitoring program for complex patients on health status, self-management, and health service utilization? The secondary research question was: What were the experiences of patients and HCPs related to the telemonitoring program and the way in which it was implemented?"

METHODS

3a) Description of trial design (such as parallel, factorial) including allocation ratio

Does your paper address CONSORT subitem 3a? *

Copy and paste relevant sections from the manuscript (include quotes in quotation marks "like this" to indicate direct quotes from your manuscript), or elaborate on this item by providing additional information not in the ms, or briefly explain why the item is not applicable/relevant for your study

"This was a pragmatic, unblinded, 1:1 randomized control trial (RCT) comparing the 6-month impact of telemonitoring to support the management of patients with complex conditions to the standard of care."

3b) Important changes to methods after trial commencement (such as eligibility criteria), with reasons

Does your paper address CONSORT subitem 3b? *

Copy and paste relevant sections from the manuscript (include quotes in quotation marks "like this" to indicate direct quotes from your manuscript), or elaborate on this item by providing additional information not in the ms, or briefly explain why the item is not applicable/relevant for your study

"Although the intent was for patients with MCCs to be followed holistically, development delays for the HT and DM modules led to HF patients being enrolled and managed for HF alone, even if they had MCCs. In addition, the siloed nature of specialty care made it such that even when the technology could support the simultaneous management of HF, DM, and HT, patients were only monitored for the condition being managed at the location of enrollment. As a result, the model of care differed depending on the structure and resources at each clinic. For example, alerts for HF patients were primarily addressed by nurse practitioners who would escalate issues to the treating cardiologist as required. In contrast, telemonitoring alert management for HT and DM was the responsibility of the treating physician."

3b-i) Bug fixes, Downtimes, Content Changes

Bug fixes, Downtimes, Content Changes: ehealth systems are often dynamic systems. A description of changes to methods therefore also includes important changes made on the intervention or comparator during the trial (e.g., major bug fixes or changes in the functionality or content) (5-iii) and other “unexpected events” that may have influenced study design such as staff changes, system failures/downtimes, etc. [2].

	1	2	3	4	5	
subitem not at all important	○	○	○	○	○	essential

Does your paper address subitem 3b-i?

Copy and paste relevant sections from the manuscript (include quotes in quotation marks "like this" to indicate direct quotes from your manuscript), or elaborate on this item by providing additional information not in the ms, or briefly explain why the item is not applicable/relevant for your study

"Although the intent was for patients with MCCs to be followed holistically, development delays for the HT and DM modules led to HF patients being enrolled and managed for HF alone, even if they had MCCs. In addition, the siloed nature of specialty care made it such that even when the technology could support the simultaneous management of HF, DM, and HT, patients were only monitored for the condition being managed at the location of enrollment. As a result, the model of care differed depending on the structure and resources at each clinic. For example, alerts for HF patients were primarily addressed by nurse practitioners who would escalate issues to the treating cardiologist as required. In contrast, telemonitoring alert management for HT and DM was the responsibility of the treating physician."

4a) Eligibility criteria for participants

Does your paper address CONSORT subitem 4a? *

Copy and paste relevant sections from the manuscript (include quotes in quotation marks "like this" to indicate direct quotes from your manuscript), or elaborate on this item by providing additional information not in the ms, or briefly explain why the item is not applicable/relevant for your study

"To be eligible, patients had to be 18 years of age or older, able to speak and read English (or have a caregiver who does), and diagnosed with HF with reduced ejection fraction (EF<40 percent), uncontrolled HT (≥140/90 mmHg auscultatory), or insulin-requiring DM and performing self-capillary glucose monitoring. Exclusion criteria included being on mechanical circulatory support, dialysis, or on the transplant list. In addition, patients with a life expectancy less than 1 year, dementia, uncontrolled psychiatric illness, or residents of a long-term care facility were excluded. See the published protocol for full criteria [33]."

4a-i) Computer / Internet literacy

Computer / Internet literacy is often an implicit “de facto” eligibility criterion - this should be explicitly clarified.

	1	2	3	4	5	
subitem not at all important	○	○	○	○	○	essential

Does your paper address subitem 4a-i?

Copy and paste relevant sections from the manuscript (include quotes in quotation marks "like this" to indicate direct quotes from your manuscript), or elaborate on this item by providing additional information not in the ms, or briefly explain why the item is not applicable/relevant for your study

Computer / internet literacy was not an eligibility criterion.

4a-ii) Open vs. closed, web-based vs. face-to-face assessments:

Open vs. closed, web-based vs. face-to-face assessments: Mention how participants were recruited (online vs. offline), e.g., from an open access website or from a clinic, and clarify if this was a purely web-based trial, or there were face-to-face components (as part of the intervention or for assessment), i.e., to what degree got the study team to know the participant. In online-only trials, clarify if participants were quasi-anonymous and whether having multiple identities was possible or whether technical or logistical measures (e.g., cookies, email confirmation, phone calls) were used to detect/prevent these.

	1	2	3	4	5	
subitem not at all important	○	○	○	○	○	essential

Does your paper address subitem 4a-ii? *

Copy and paste relevant sections from the manuscript (include quotes in quotation marks "like this" to indicate direct quotes from your manuscript), or elaborate on this item by providing additional information not in the ms, or briefly explain why the item is not applicable/relevant for your study

"Patients with HF were recruited from the Heart Function Clinic at the University Health Network (UHN), a large academic hospital in Toronto, Canada, between August 2016 and February 2018. Patients with HT were recruited from a HT clinic at Mount Sinai Hospital between July-December 2019 and DM patients were recruited from the UHN Endocrinology Clinic between August-December 2019. The study received approval from the UHN (15-9995-BE) and Mount Sinai Hospital (MSH REB 16-0093-E) Research Ethics Boards."

4a-iii) Information giving during recruitment

Information given during recruitment. Specify how participants were briefed for recruitment and in the informed consent procedures (e.g., publish the informed consent documentation as appendix, see also item X26), as this information may have an effect on user self-selection, user expectation and may also bias results.

	1	2	3	4	5	
subitem not at all important	○	○	○	○	○	essential

Does your paper address subitem 4a-iii?

Copy and paste relevant sections from the manuscript (include quotes in quotation marks "like this" to indicate direct quotes from your manuscript), or elaborate on this item by providing additional information not in the ms, or briefly explain why the item is not applicable/relevant for your study

"Patients with HF were instructed to monitor daily weight, blood pressure, heart rate, and symptoms. Due to the frequency of readings and higher complexity of the HF Medly algorithm [36], patient feedback was designed to be highly actionable. For example, patients were told to take a dose of their prescribed diuretic and restrict salts and fluids upon a large weight gain. Patients with DM were instructed to record blood glucose readings at least once per week or more as instructed by their HCP and received actionable feedback (e.g., “Eat 15g (1 tbls) of sugar or other fast-acting carbohydrates” in response to low blood glucose readings). Patients with HT were instructed to report their blood pressure once every two weeks unless the readings were out of range, in which case, they would be instructed through the app to increase the frequency of their readings. All modules had messages instructing users to contact the clinic or to go to the emergency department when critical parameters were out of range. To assist with adherence, an automated phone call was sent to patients based on the required frequency of each condition’s algorithm."

4b) Settings and locations where the data were collected

Does your paper address CONSORT subitem 4b? *

Copy and paste relevant sections from the manuscript (include quotes in quotation marks "like this" to indicate direct quotes from your manuscript), or elaborate on this item by providing additional information not in the ms, or briefly explain why the item is not applicable/relevant for your study

"This was a pragmatic, unblinded, 1:1 randomized control trial (RCT) comparing the 6-month impact of telemonitoring to support the management of patients with complex conditions to the standard of care. An embedded qualitative component was included to understand the results of the trial. Patients with HF were recruited from the Heart Function Clinic at the University Health Network (UHN), a large academic hospital in Toronto, Canada, between August 2016 and February 2018. Patients with HT were recruited from a HT clinic at Mount Sinai Hospital between July-December 2019 and DM patients were recruited from the UHN Endocrinology Clinic between August-December 2019. The study received approval from the UHN (15-9995-BE) and Mount Sinai Hospital (MSH REB 16-0093-E) Research Ethics Boards."

4b-i) Report if outcomes were (self-)assessed through online questionnaires

Clearly report if outcomes were (self-)assessed through online questionnaires (as common in web-based trials) or otherwise.

	1	2	3	4	5	
subitem not at all important	○	○	○	○	○	essential

Does your paper address subitem 4b-i? *

Copy and paste relevant sections from the manuscript (include quotes in quotation marks "like this" to indicate direct quotes from your manuscript), or elaborate on this item by providing additional information not in the ms, or briefly explain why the item is not applicable/relevant for your study

Outcomes were not self-assessed through online questionnaires

4b-ii) Report how institutional affiliations are displayed

Report how institutional affiliations are displayed to potential participants [on ehealth media], as affiliations with prestigious hospitals or universities may affect volunteer rates, use, and reactions with regards to an intervention.(Not a required item – describe only if this may bias results)

	1	2	3	4	5	
subitem not at all important	○	○	○	○	○	essential

Does your paper address subitem 4b-ii?

Copy and paste relevant sections from the manuscript (include quotes in quotation marks "like this" to indicate direct quotes from your manuscript), or elaborate on this item by providing additional information not in the ms, or briefly explain why the item is not applicable/relevant for your study

Your answer

5) The interventions for each group with sufficient details to allow replication, including how and when they were actually administered

5-i) Mention names, credential, affiliations of the developers, sponsors, and owners

Mention names, credential, affiliations of the developers, sponsors, and owners [6] (if authors/evaluators are owners or developer of the software, this needs to be declared in a “Conflict of interest” section or mentioned elsewhere in the manuscript).

	1	2	3	4	5	
subitem not at all important	○	○	○	○	○	essential

Does your paper address subitem 5-i?

Copy and paste relevant sections from the manuscript (include quotes in quotation marks "like this" to indicate direct quotes from your manuscript), or elaborate on this item by providing additional information not in the ms, or briefly explain why the item is not applicable/relevant for your study

"ES, HR, JC are considered inventors of the Medly system under the intellectual property policies of the UHN and may benefit from future commercialization of the technology by UHN."

5-ii) Describe the history/development process

Describe the history/development process of the application and previous formative evaluations (e.g., focus groups, usability testing), as these will have an impact on adoption/use rates and help with interpreting results.

	1	2	3	4	5	
subitem not at all important	○	○	○	○	○	essential

Does your paper address subitem 5-ii?

Copy and paste relevant sections from the manuscript (include quotes in quotation marks "like this" to indicate direct quotes from your manuscript), or elaborate on this item by providing additional information not in the ms, or briefly explain why the item is not applicable/relevant for your study

"The intervention was a mobile phone-based telemonitoring program involving a system, named Medly, which enables patients with chronic conditions to take relevant physiological measurements with wireless home medical devices and to answer symptom questions using the Medly smartphone app. In response to these inputs, rule-based algorithms which were iteratively developed and validated by HF, DM, and HT specialists [34] and customized through target thresholds, displayed self-care instructions to patients (Figure 1) and sent alerts to the clinical team via email and a secure web portal where historical trends could also be viewed [34]. As such, the system was designed to improve patient self-management and provide clinical decision support to HCPs [35]."

5-iii) Revisions and updating

Revisions and updating. Clearly mention the date and/or version number of the application/intervention (and comparator, if applicable) evaluated, or describe whether the intervention underwent major changes during the evaluation process, or whether the development and/or content was "frozen" during the trial. Describe dynamic components such as news feeds or changing content which may have an impact on the replicability of the intervention (for unexpected events see item 3b).

	1	2	3	4	5	
subitem not at all important	○	○	○	○	○	essential

Does your paper address subitem 5-iii?

Copy and paste relevant sections from the manuscript (include quotes in quotation marks "like this" to indicate direct quotes from your manuscript), or elaborate on this item by providing additional information not in the ms, or briefly explain why the item is not applicable/relevant for your study

N/A - No revisions were made to the application during the evaluation process.

5-iv) Quality assurance methods

Provide information on quality assurance methods to ensure accuracy and quality of information provided [1], if applicable.

	1	2	3	4	5	
subitem not at all important	○	○	○	○	○	essential

Does your paper address subitem 5-iv?

Copy and paste relevant sections from the manuscript (include quotes in quotation marks "like this" to indicate direct quotes from your manuscript), or elaborate on this item by providing additional information not in the ms, or briefly explain why the item is not applicable/relevant for your study

Your answer

5-v) Ensure replicability by publishing the source code, and/or providing screenshots/screen-capture video, and/or providing flowcharts of the algorithms used

Ensure replicability by publishing the source code, and/or providing screenshots/screen-capture video, and/or providing flowcharts of the algorithms used. Replicability (i.e., other researchers should in principle be able to replicate the study) is a hallmark of scientific reporting.

	1	2	3	4	5	
subitem not at all important	○	○	○	○	○	essential

Does your paper address subitem 5-v?

Copy and paste relevant sections from the manuscript (include quotes in quotation marks "like this" to indicate direct quotes from your manuscript), or elaborate on this item by providing additional information not in the ms, or briefly explain why the item is not applicable/relevant for your study

"In response to these inputs, rule-based algorithms which were iteratively developed and validated by HF, DM, and HT specialists [34] and customized through target thresholds, displayed self-care instructions to patients (Figure 1) and sent alerts to the clinical team via email and a secure web portal where historical trends could also be viewed [34]."

5-vi) Digital preservation

Digital preservation: Provide the URL of the application, but as the intervention is likely to change or disappear over the course of the years; also make sure the intervention is archived (Internet Archive, webcitation.org, and/or publishing the source code or screenshots/videos alongside the article). As pages behind login screens cannot be archived, consider creating demo pages which are accessible without login.

	1	2	3	4	5	
subitem not at all important	○	○	○	○	○	essential

Does your paper address subitem 5-vi?

Copy and paste relevant sections from the manuscript (include quotes in quotation marks "like this" to indicate direct quotes from your manuscript), or elaborate on this item by providing additional information not in the ms, or briefly explain why the item is not applicable/relevant for your study

The Medly system is available by prescription only and is not available to the public. Screenshots of the intervention have been provided in Figure 1.

5-vii) Access

Access: Describe how participants accessed the application, in what setting/context, if they had to pay (or were paid) or not, whether they had to be a member of specific group. If known, describe how participants obtained “access to the platform and Internet” [1]. To ensure access for editors/reviewers/readers, consider to provide a “backdoor” login account or demo mode for reviewers/readers to explore the application (also important for archiving purposes, see vi).

	1	2	3	4	5	
subitem not at all important	○	○	○	○	○	essential

Does your paper address subitem 5-vii? *

Copy and paste relevant sections from the manuscript (include quotes in quotation marks "like this" to indicate direct quotes from your manuscript), or elaborate on this item by providing additional information not in the ms, or briefly explain why the item is not applicable/relevant for your study

"The intervention was a mobile phone-based telemonitoring program involving a system, named Medly, which enables patients with chronic conditions to take relevant physiological measurements with wireless home medical devices and to answer symptom questions using the Medly smartphone app. In response to these inputs, rule-based algorithms which were iteratively developed and validated by HF, DM, and HT specialists [34] and customized through target thresholds, displayed self-care instructions to patients (Figure 1) and sent alerts to the clinical team via email and a secure web portal where historical trends could also be viewed [34]. As such, the system was designed to improve patient self-management and provide clinical decision support to HCPs [35]. Participants were provided with the necessary equipment, including a smartphone and relevant Bluetooth devices (weight scale, blood pressure monitoring, blood glucose monitor) [33]."

5-viii) Mode of delivery, features/functionalities/components of the intervention and comparator, and the theoretical framework

Describe mode of delivery, features/functionalities/components of the intervention and comparator, and the theoretical framework [6] used to design them (instructional strategy [1], behaviour change techniques, persuasive features, etc., see e.g., [7, 8] for terminology). This includes an in-depth description of the content (including where it is coming from and who developed it) [1]," whether [and how] it is tailored to individual circumstances and allows users to track their progress and receive feedback" [6]. This also includes a description of communication delivery channels and – if computer-mediated communication is a component – whether communication was synchronous or asynchronous [6]. It also includes information on presentation strategies [1], including page design principles, average amount of text on pages, presence of hyperlinks to other resources, etc. [1].

	1	2	3	4	5	
subitem not at all important	○	○	○	○	○	essential

Does your paper address subitem 5-viii? *

Copy and paste relevant sections from the manuscript (include quotes in quotation marks "like this" to indicate direct quotes from your manuscript), or elaborate on this item by providing additional information not in the ms, or briefly explain why the item is not applicable/relevant for your study

"The intervention was a mobile phone-based telemonitoring program involving a system, named Medly, which enables patients with chronic conditions to take relevant physiological measurements with wireless home medical devices and to answer symptom questions using the Medly smartphone app. In response to these inputs, rule-based algorithms which were iteratively developed and validated by HF, DM, and HT specialists [34] and customized through target thresholds, displayed self-care instructions to patients (Figure 1) and sent alerts to the clinical team via email and a secure web portal where historical trends could also be viewed [34]. As such, the system was designed to improve patient self-management and provide clinical decision support to HCPs [35]. Participants were provided with the necessary equipment, including a smartphone and relevant Bluetooth devices (weight scale, blood pressure monitoring, blood glucose monitor) [33]."

5-ix) Describe use parameters

Describe use parameters (e.g., intended “doses” and optimal timing for use). Clarify what instructions or recommendations were given to the user, e.g., regarding timing, frequency, heaviness of use, if any, or was the intervention used ad libitum.

	1	2	3	4	5	
subitem not at all important	○	○	○	○	○	essential

Does your paper address subitem 5-ix?

Copy and paste relevant sections from the manuscript (include quotes in quotation marks "like this" to indicate direct quotes from your manuscript), or elaborate on this item by providing additional information not in the ms, or briefly explain why the item is not applicable/relevant for your study

"Patients with DM were instructed to record blood glucose readings at least once per week or more as instructed by their HCP and received actionable feedback (e.g., “Eat 15g (1 tbls) of sugar or other fast-acting carbohydrates” in response to low blood glucose readings). Patients with HT were instructed to report their blood pressure once every two weeks unless the readings were out of range, in which case, they would be instructed through the app to increase the frequency of their readings. All modules had messages instructing users to contact the clinic or to go to the emergency department when critical parameters were out of range. To assist with adherence, an automated phone call was sent to patients based on the required frequency of each condition’s algorithm."

5-x) Clarify the level of human involvement

Clarify the level of human involvement (care providers or health professionals, also technical assistance) in the e-intervention or as co-intervention (detail number and expertise of professionals involved, if any, as well as “type of assistance offered, the timing and frequency of the support, how it is initiated, and the medium by which the assistance is delivered”. It may be necessary to distinguish between the level of human involvement required for the trial, and the level of human involvement required for a routine application outside of a RCT setting (discuss under item 21 – generalizability).

	1	2	3	4	5	
subitem not at all important	○	○	○	○	○	essential

Does your paper address subitem 5-x?

Copy and paste relevant sections from the manuscript (include quotes in quotation marks "like this" to indicate direct quotes from your manuscript), or elaborate on this item by providing additional information not in the ms, or briefly explain why the item is not applicable/relevant for your study

"In addition, the siloed nature of specialty care made it such that even when the technology could support the simultaneous management of HF, DM, and HT, patients were only monitored for the condition being managed at the location of enrollment. As a result, the model of care differed depending on the structure and resources at each clinic. For example, alerts for HF patients were primarily addressed by nurse practitioners who would escalate issues to the treating cardiologist as required. In contrast, telemonitoring alert management for HT and DM was the responsibility of the treating physician."

5-xi) Report any prompts/reminders used

Report any prompts/reminders used: Clarify if there were prompts (letters, emails, phone calls, SMS) to use the application, what triggered them, frequency etc. It may be necessary to distinguish between the level of prompts/reminders required for the trial, and the level of prompts/reminders for a routine application outside of a RCT setting (discuss under item 21 – generalizability).

	1	2	3	4	5	
subitem not at all important	○	○	○	○	○	essential

Does your paper address subitem 5-xi? *

Copy and paste relevant sections from the manuscript (include quotes in quotation marks "like this" to indicate direct quotes from your manuscript), or elaborate on this item by providing additional information not in the ms, or briefly explain why the item is not applicable/relevant for your study

"To assist with adherence, an automated phone call was sent to patients based on the required frequency of each condition's algorithm."

5-xii) Describe any co-interventions (incl. training/support)

Describe any co-interventions (incl. training/support): Clearly state any interventions that are provided in addition to the targeted eHealth intervention, as ehealth intervention may not be designed as stand-alone intervention. This includes training sessions and support [1]. It may be necessary to distinguish between the level of training required for the trial, and the level of training for a routine application outside of a RCT setting (discuss under item 21 – generalizability.

	1	2	3	4	5	
subitem not at all important	○	○	○	○	○	essential

Does your paper address subitem 5-xii? *

Copy and paste relevant sections from the manuscript (include quotes in quotation marks "like this" to indicate direct quotes from your manuscript), or elaborate on this item by providing additional information not in the ms, or briefly explain why the item is not applicable/relevant for your study

"Patients allocated to the intervention arm were provided with the telemonitoring equipment and user manual before receiving face-to-face training on how to use the equipment."

6a) Completely defined pre-specified primary and secondary outcome measures, including how and when they were assessed

Does your paper address CONSORT subitem 6a? *

Copy and paste relevant sections from the manuscript (include quotes in quotation marks "like this" to indicate direct quotes from your manuscript), or elaborate on this item by providing additional information not in the ms, or briefly explain why the item is not applicable/relevant for your study

"The primary outcome was health status as measured by the SF-36. Secondary outcomes included anxiety and depression as measured with the Hospital Anxiety and Depression Scale (HADS)[40], patients' self-efficacy to manage their condition as measured with the Self-Efficacy for Managing Chronic Disease 6-Item scale (SEMCD6)[41–43], and the number of self-reported interactions with the health system in the previous six months were also collected, including hospitalizations, ED visits, visits to specialty care clinics, and visits to family doctors. Self-care as measured by the Self-Care of Heart Failure Index (SCHFI)[44] and HF-specific quality of life as measured by Minnesota Living with Heart Failure Questionnaire (MLHFQ)[45] were also collected for HF patients."

"Questionnaires containing the patient-reported outcome measures were administered at baseline and 6 months [33]. Demographic questions were included in the baseline questionnaire to characterize the study participants. Patient adherence was calculated from Medly server log data as a percentage of completed recommended readings over the course of the 6-month trial."

6a-i) Online questionnaires: describe if they were validated for online use and apply CHERRIES items to describe how the questionnaires were designed/deployed

If outcomes were obtained through online questionnaires, describe if they were validated for online use and apply CHERRIES items to describe how the questionnaires were designed/deployed [9].

	1	2	3	4	5	
subitem not at all important	○	○	○	○	○	essential

Does your paper address subitem 6a-i?

Copy and paste relevant sections from manuscript text

N/A - Outcomes were obtained through mailed questionnaires.

6a-ii) Describe whether and how “use” (including intensity of use/dosage) was defined/measured/monitored

Describe whether and how “use” (including intensity of use/dosage) was defined/measured/monitored (logins, logfile analysis, etc.). Use/adoption metrics are important process outcomes that should be reported in any ehealth trial.

	1	2	3	4	5	
subitem not at all important	○	○	○	○	○	essential

Does your paper address subitem 6a-ii?

Copy and paste relevant sections from manuscript text

"Patients with HF were instructed to monitor daily weight, blood pressure, heart rate, and symptoms. Due to the frequency of readings and higher complexity of the HF Medly algorithm [36], patient feedback was designed to be highly actionable. For example, patients were told to take a dose of their prescribed diuretic and restrict salts and fluids upon a large weight gain. Patients with DM were instructed to record blood glucose readings at least once per week or more as instructed by their HCP and received actionable feedback (e.g., “Eat 15g (1 tbls) of sugar or other fast-acting carbohydrates” in response to low blood glucose readings). Patients with HT were instructed to report their blood pressure once every two weeks unless the readings were out of range, in which case, they would be instructed through the app to increase the frequency of their readings."

"Patient adherence was calculated from Medly server log data as a percentage of completed recommended readings over the course of the 6-month trial."

"Of the 41 patients who completed the study in the intervention arm, 29 patients used the HF module, 10 patients used the HT module, and 2 patients used the DM module. On average, HF patients completed all 4 readings 78.1% of the days they were enrolled in the 6 month trials. This usage rate increased to 86.2% when looking at the percentage of days HF patients reported at least one reading. Average adherence was 64.2% for the HT patients and 58.3% for DM patients based on a minimum of biweekly and weekly readings for HT and DM, respectively. The combined average adherence across all subjects was 73.1% over the study period. Figure 3 depicts the monthly adherence rates for the 3 disease modules. It shows that although adherence was initially high across all conditions and remained relatively stable for HF patients, it dropped markedly after the second month for HT and DM [illegible]"

6a-iii) Describe whether, how, and when qualitative feedback from participants was obtained

Describe whether, how, and when qualitative feedback from participants was obtained (e.g., through emails, feedback forms, interviews, focus groups).

	1	2	3	4	5	
subitem not at all important	○	○	○	○	○	essential

Does your paper address subitem 6a-iii?

Copy and paste relevant sections from manuscript text

"All HCPs who used the system and a sample of patients in the intervention group were invited to participate in post-study semi structured interviews which probed their experience in the telemonitoring program. Patients were identified through a convenience sample with efforts made to include patients from the 3 conditions monitored. Interviews took place in a private clinic room or over the telephone and were audio recorded for later transcription.

Qualitative data were analyzed using a conventional content analysis approach [46] by two researchers who first analyzed the transcripts independently and then met to discuss themes until consensus was reached. NVivo version 11 (QSR International) was used to help organize the themes."

6b) Any changes to trial outcomes after the trial commenced, with reasons

Does your paper address CONSORT subitem 6b? *

Copy and paste relevant sections from the manuscript (include quotes in quotation marks "like this" to indicate direct quotes from your manuscript), or elaborate on this item by providing additional information not in the ms, or briefly explain why the item is not applicable/relevant for your study

No changes were made to the trial outcomes after the trial commenced.

7a) How sample size was determined

NPT: When applicable, details of whether and how the clustering by care provides or centers was addressed

7a-i) Describe whether and how expected attrition was taken into account when calculating the sample size

Describe whether and how expected attrition was taken into account when calculating the sample size.

	1	2	3	4	5	
subitem not at all important	○	○	○	○	○	essential

Does your paper address subitem 7a-i?

Copy and paste relevant sections from manuscript title (include quotes in quotation marks "like this" to indicate direct quotes from your manuscript), or elaborate on this item by providing additional information not in the ms, or briefly explain why the item is not applicable/relevant for your study

"A total of 146 patients of varying chronic conditions were targeted for enrolment (see sample size justification [33])."

Protocol outlines how attrition was taken into account when calculating the sample size.

7b) When applicable, explanation of any interim analyses and stopping guidelines

Does your paper address CONSORT subitem 7b? *

Copy and paste relevant sections from the manuscript (include quotes in quotation marks "like this" to indicate direct quotes from your manuscript), or elaborate on this item by providing additional information not in the ms, or briefly explain why the item is not applicable/relevant for your study

No interim analyses and stopping guidelines were applied in this study.

8a) Method used to generate the random allocation sequence

NPT: When applicable, how care providers were allocated to each trial group

Does your paper address CONSORT subitem 8a? *

Copy and paste relevant sections from the manuscript (include quotes in quotation marks "like this" to indicate direct quotes from your manuscript), or elaborate on this item by providing additional information not in the ms, or briefly explain why the item is not applicable/relevant for your study

"Patients were then blocked randomized using blocks of 4 into the intervention and standard of care treatments groups in a 1:1 ratio as per the published stratification protocol [33]."

8b) Type of randomisation; details of any restriction (such as blocking and block size)

Does your paper address CONSORT subitem 8b? *

Copy and paste relevant sections from the manuscript (include quotes in quotation marks "like this" to indicate direct quotes from your manuscript), or elaborate on this item by providing additional information not in the ms, or briefly explain why the item is not applicable/relevant for your study

"Patients were then blocked randomized using blocks of 4 into the intervention and standard of care treatments groups in a 1:1 ratio as per the published stratification protocol [33]."

9) Mechanism used to implement the random allocation sequence (such as sequentially numbered containers), describing any steps taken to conceal the sequence until interventions were assigned

Does your paper address CONSORT subitem 9? *

Copy and paste relevant sections from the manuscript (include quotes in quotation marks "like this" to indicate direct quotes from your manuscript), or elaborate on this item by providing additional information not in the ms, or briefly explain why the item is not applicable/relevant for your study

Described in the published protocol referenced in the manuscript.

10) Who generated the random allocation sequence, who enrolled participants, and who assigned participants to interventions

Does your paper address CONSORT subitem 10? *

Copy and paste relevant sections from the manuscript (include quotes in quotation marks "like this" to indicate direct quotes from your manuscript), or elaborate on this item by providing additional information not in the ms, or briefly explain why the item is not applicable/relevant for your study

Described in the published protocol referenced in the manuscript.

11a) If done, who was blinded after assignment to interventions (for example, participants, care providers, those assessing outcomes) and how

NPT: Whether or not administering co-interventions were blinded to group assignment

11a-i) Specify who was blinded, and who wasn’t

Specify who was blinded, and who wasn’t. Usually, in web-based trials it is not possible to blind the participants [1, 3] (this should be clearly acknowledged), but it may be possible to blind outcome assessors, those doing data analysis or those administering co-interventions (if any).

	1	2	3	4	5	
subitem not at all important	○	○	○	○	○	essential

Does your paper address subitem 11a-i? *

Copy and paste relevant sections from the manuscript (include quotes in quotation marks "like this" to indicate direct quotes from your manuscript), or elaborate on this item by providing additional information not in the ms, or briefly explain why the item is not applicable/relevant for your study

Described in the published protocol referenced in the manuscript.

11a-ii) Discuss e.g., whether participants knew which intervention was the “intervention of interest” and which one was the “comparator”

Informed consent procedures (4a-ii) can create biases and certain expectations - discuss e.g., whether participants knew which intervention was the “intervention of interest” and which one was the “comparator”.

	1	2	3	4	5	
subitem not at all important	○	○	○	○	○	essential

Does your paper address subitem 11a-ii?

Copy and paste relevant sections from the manuscript (include quotes in quotation marks "like this" to indicate direct quotes from your manuscript), or elaborate on this item by providing additional information not in the ms, or briefly explain why the item is not applicable/relevant for your study

Described in the published protocol referenced in the manuscript.

11b) If relevant, description of the similarity of interventions

(this item is usually not relevant for ehealth trials as it refers to similarity of a placebo or sham intervention to a active medication/intervention)

Does your paper address CONSORT subitem 11b? *

Copy and paste relevant sections from the manuscript (include quotes in quotation marks "like this" to indicate direct quotes from your manuscript), or elaborate on this item by providing additional information not in the ms, or briefly explain why the item is not applicable/relevant for your study

N/A - Trial compared telemonitoring to standard of care (seeing the clinical team for scheduled follow-ups every 3-6 months, optimization of medical therapy, and self-management education).

12a) Statistical methods used to compare groups for primary and secondary outcomes

NPT: When applicable, details of whether and how the clustering by care providers or centers was addressed

Does your paper address CONSORT subitem 12a? *

Copy and paste relevant sections from the manuscript (include quotes in quotation marks "like this" to indicate direct quotes from your manuscript), or elaborate on this item by providing additional information not in the ms, or briefly explain why the item is not applicable/relevant for your study

"Questionnaires containing the patient-reported outcome measures were administered at baseline and 6 months [33]. Demographic questions were included in the baseline questionnaire to characterize the study participants. Patient adherence was calculated from Medly server log data as a percentage of completed recommended readings over the course of the 6-month trial.

Post-trial data and change scores were compared between the treatment arms using independent Student t tests and Mann–Whitney tests (for normally and not normally distributed data, respectively). Paired Student t tests and Wilcoxon signed rank tests were performed to compare baseline and post-study data within the control and telemonitoring groups. Analyses were performed using IBM SPSS (Version 27) under the intention-to-treat principle and using a significance P<.05."

12a-i) Imputation techniques to deal with attrition / missing values

Imputation techniques to deal with attrition / missing values: Not all participants will use the intervention/comparator as intended and attrition is typically high in ehealth trials. Specify how participants who did not use the application or dropped out from the trial were treated in the statistical analysis (a complete case analysis is strongly discouraged, and simple imputation techniques such as LOCF may also be problematic [4]).

	1	2	3	4	5	
subitem not at all important	○	○	○	○	○	essential

Does your paper address subitem 12a-i? *

Copy and paste relevant sections from the manuscript (include quotes in quotation marks "like this" to indicate direct quotes from your manuscript), or elaborate on this item by providing additional information not in the ms, or briefly explain why the item is not applicable/relevant for your study

"Therefore, although 45 and 50 patients were allocated to telemonitoring and control arms, only 28 and 26 complete data sets were available for analysis in the telemonitoring and control arms, respectively."

12b) Methods for additional analyses, such as subgroup analyses and adjusted analyses

Does your paper address CONSORT subitem 12b? *

Copy and paste relevant sections from the manuscript (include quotes in quotation marks "like this" to indicate direct quotes from your manuscript), or elaborate on this item by providing additional information not in the ms, or briefly explain why the item is not applicable/relevant for your study

N/A - No additional analyses were conducted.

X26) REB/IRB Approval and Ethical Considerations [recommended as subheading under "Methods"] (not a CONSORT item)

X26-i) Comment on ethics committee approval

	1	2	3	4	5	
subitem not at all important	○	○	○	○	○	essential

Does your paper address subitem X26-i?

Copy and paste relevant sections from the manuscript (include quotes in quotation marks "like this" to indicate direct quotes from your manuscript), or elaborate on this item by providing additional information not in the ms, or briefly explain why the item is not applicable/relevant for your study

"The study received approval from the UHN (15-9995-BE) and Mount Sinai Hospital (MSH REB 16-0093-E) Research Ethics Boards."

x26-ii) Outline informed consent procedures

Outline informed consent procedures e.g., if consent was obtained offline or online (how? Checkbox, etc.?), and what information was provided (see 4a-ii). See [6] for some items to be included in informed consent documents.

	1	2	3	4	5	
subitem not at all important	○	○	○	○	○	essential

Does your paper address subitem X26-ii?

Copy and paste relevant sections from the manuscript (include quotes in quotation marks "like this" to indicate direct quotes from your manuscript), or elaborate on this item by providing additional information not in the ms, or briefly explain why the item is not applicable/relevant for your study

"After confirming eligibility, the coordinator explained the study and obtained informed consent."

Consent procedures also described in referenced protocol.

X26-iii) Safety and security procedures

Safety and security procedures, incl. privacy considerations, and any steps taken to reduce the likelihood or detection of harm (e.g., education and training, availability of a hotline)

	1	2	3	4	5	
subitem not at all important	○	○	○	○	○	essential

Does your paper address subitem X26-iii?

Copy and paste relevant sections from the manuscript (include quotes in quotation marks "like this" to indicate direct quotes from your manuscript), or elaborate on this item by providing additional information not in the ms, or briefly explain why the item is not applicable/relevant for your study

"Patients allocated to the intervention arm were provided with the telemonitoring equipment and user manual before receiving face-to-face training on how to use the equipment."

RESULTS

13a) For each group, the numbers of participants who were randomly assigned, received intended treatment, and were analysed for the primary outcome

NPT: The number of care providers or centers performing the intervention in each group and the number of patients treated by each care provider in each center

Does your paper address CONSORT subitem 13a? *

Copy and paste relevant sections from the manuscript (include quotes in quotation marks "like this" to indicate direct quotes from your manuscript), or elaborate on this item by providing additional information not in the ms, or briefly explain why the item is not applicable/relevant for your study

"Figure 2. Flow of patient participants through the trial."

13b) For each group, losses and exclusions after randomisation, together with reasons

Does your paper address CONSORT subitem 13b? (NOTE: Preferably, this is shown in a CONSORT flow diagram) *

Copy and paste relevant sections from the manuscript (include quotes in quotation marks "like this" to indicate direct quotes from your manuscript), or elaborate on this item by providing additional information not in the ms, or briefly explain why the item is not applicable/relevant for your study

"Figure 2. Flow of patient participants through the trial."

13b-i) Attrition diagram

Strongly recommended: An attrition diagram (e.g., proportion of participants still logging in or using the intervention/comparator in each group plotted over time, similar to a survival curve) or other figures or tables demonstrating usage/dose/engagement.

	1	2	3	4	5	
subitem not at all important	○	○	○	○	○	essential

Does your paper address subitem 13b-i?

Copy and paste relevant sections from the manuscript or cite the figure number if applicable (include quotes in quotation marks "like this" to indicate direct quotes from your manuscript), or elaborate on this item by providing additional information not in the ms, or briefly explain why the item is not applicable/relevant for your study

"Figure 3 depicts the monthly adherence rates for the 3 disease modules. It shows that although adherence was initially high across all conditions and remained relatively stable for HF patients, it dropped markedly after the second month for HT and DM patients."

14a) Dates defining the periods of recruitment and follow-up

Does your paper address CONSORT subitem 14a? *

Copy and paste relevant sections from the manuscript (include quotes in quotation marks "like this" to indicate direct quotes from your manuscript), or elaborate on this item by providing additional information not in the ms, or briefly explain why the item is not applicable/relevant for your study

"Patients with HT were recruited from a HT clinic at Mount Sinai Hospital between July-December 2019 and DM patients were recruited from the UHN Endocrinology Clinic between August-December 2019."

14a-i) Indicate if critical “secular events” fell into the study period

Indicate if critical “secular events” fell into the study period, e.g., significant changes in Internet resources available or “changes in computer hardware or Internet delivery resources”

	1	2	3	4	5	
subitem not at all important	○	○	○	○	○	essential

Does your paper address subitem 14a-i?

Copy and paste relevant sections from the manuscript (include quotes in quotation marks "like this" to indicate direct quotes from your manuscript), or elaborate on this item by providing additional information not in the ms, or briefly explain why the item is not applicable/relevant for your study

"Finally, and most importantly, the COVID-19 pandemic led to a freeze to non-essential research activities and a significant shift toward virtual care which fundamentally altered the control group after the research freeze was lifted.

In addition to ending recruitment, shifting priorities at the onset of COVID-19 also impacted the collection of post-study data as patients followed for HT and DM ended their enrollment during the first wave, which led to a higher rate of incomplete questionnaires."

14b) Why the trial ended or was stopped (early)

Does your paper address CONSORT subitem 14b? *

Copy and paste relevant sections from the manuscript (include quotes in quotation marks "like this" to indicate direct quotes from your manuscript), or elaborate on this item by providing additional information not in the ms, or briefly explain why the item is not applicable/relevant for your study

"Finally, and most importantly, the COVID-19 pandemic led to a freeze to non-essential research activities and a significant shift toward virtual care which fundamentally altered the control group after the research freeze was lifted. "

15) A table showing baseline demographic and clinical characteristics for each group

NPT: When applicable, a description of care providers (case volume, qualification, expertise, etc.) and centers (volume) in each group

Does your paper address CONSORT subitem 15? *

Copy and paste relevant sections from the manuscript (include quotes in quotation marks "like this" to indicate direct quotes from your manuscript), or elaborate on this item by providing additional information not in the ms, or briefly explain why the item is not applicable/relevant for your study

"Study participants were predominantly male (54/96, 76.1%) and had an average age of 59 (SD 12.6) years. These, along with the other demographics presented in Table 1, are representative of the UHN Heart Function Clinic where most of the patient participants were recruited."

15-i) Report demographics associated with digital divide issues

In ehealth trials it is particularly important to report demographics associated with digital divide issues, such as age, education, gender, social-economic status, computer/Internet/ehealth literacy of the participants, if known.

	1	2	3	4	5	
subitem not at all important	○	○	○	○	○	essential

Does your paper address subitem 15-i? *

Copy and paste relevant sections from the manuscript (include quotes in quotation marks "like this" to indicate direct quotes from your manuscript), or elaborate on this item by providing additional information not in the ms, or briefly explain why the item is not applicable/relevant for your study

"Table 1. Baseline characteristics of patient participants"

16) For each group, number of participants (denominator) included in each analysis and whether the analysis was by original assigned groups

16-i) Report multiple “denominators” and provide definitions

Report multiple “denominators” and provide definitions: Report N’s (and effect sizes) “across a range of study participation [and use] thresholds” [1], e.g., N exposed, N consented, N used more than x times, N used more than y weeks, N participants “used” the intervention/comparator at specific pre-defined time points of interest (in absolute and relative numbers per group). Always clearly define “use” of the intervention.

	1	2	3	4	5	
subitem not at all important	○	○	○	○	○	essential

Does your paper address subitem 16-i? *

Copy and paste relevant sections from the manuscript (include quotes in quotation marks "like this" to indicate direct quotes from your manuscript), or elaborate on this item by providing additional information not in the ms, or briefly explain why the item is not applicable/relevant for your study

"Table 2. Independent Student t test for SF-36, HADS, Self-Efficacy for Managing Chronic Disease Scale, self-reported utilization, and Minnesota Living with Heart Failure Questionnaire, and Self-Care of Heart Failure Index"

16-ii) Primary analysis should be intent-to-treat

Primary analysis should be intent-to-treat, secondary analyses could include comparing only “users”, with the appropriate caveats that this is no longer a randomized sample (see 18-i).

	1	2	3	4	5	
subitem not at all important	○	○	○	○	○	essential

Does your paper address subitem 16-ii?

Copy and paste relevant sections from the manuscript (include quotes in quotation marks "like this" to indicate direct quotes from your manuscript), or elaborate on this item by providing additional information not in the ms, or briefly explain why the item is not applicable/relevant for your study

"Analyses were performed using IBM SPSS (Version 27) under the intention-to-treat principle and using a significance P<.05."

17a) For each primary and secondary outcome, results for each group, and the estimated effect size and its precision (such as 95% confidence interval)

Does your paper address CONSORT subitem 17a? *

Copy and paste relevant sections from the manuscript (include quotes in quotation marks "like this" to indicate direct quotes from your manuscript), or elaborate on this item by providing additional information not in the ms, or briefly explain why the item is not applicable/relevant for your study

"Table 2. Independent Student t test for SF-36, HADS, Self-Efficacy for Managing Chronic Disease Scale, self-reported utilization, and Minnesota Living with Heart Failure Questionnaire, and Self-Care of Heart Failure Index"

17a-i) Presentation of process outcomes such as metrics of use and intensity of use

In addition to primary/secondary (clinical) outcomes, the presentation of process outcomes such as metrics of use and intensity of use (dose, exposure) and their operational definitions is critical. This does not only refer to metrics of attrition (13-b) (often a binary variable), but also to more continuous exposure metrics such as “average session length”. These must be accompanied by a technical description how a metric like a “session” is defined (e.g., timeout after idle time) [1] (report under item 6a).

	1	2	3	4	5	
subitem not at all important	○	○	○	○	○	essential

Does your paper address subitem 17a-i?

Copy and paste relevant sections from the manuscript (include quotes in quotation marks "like this" to indicate direct quotes from your manuscript), or elaborate on this item by providing additional information not in the ms, or briefly explain why the item is not applicable/relevant for your study

"Of the 41 patients who completed the study in the intervention arm, 29 patients used the HF module, 10 patients used the HT module, and 2 patients used the DM module. On average, HF patients completed all 4 readings 78.1% of the days they were enrolled in the 6 month trials. This usage rate increased to 86.2% when looking at the percentage of days HF patients reported at least one reading. Average adherence was 64.2% for the HT patients and 58.3% for DM patients based on a minimum of biweekly and weekly readings for HT and DM, respectively. The combined average adherence across all subjects was 73.1% over the study period. Figure 3 depicts the monthly adherence rates for the 3 disease modules. It shows that although adherence was initially high across all conditions and remained relatively stable for HF patients, it dropped markedly after the second month for HT and DM patients."

17b) For binary outcomes, presentation of both absolute and relative effect sizes is recommended

Does your paper address CONSORT subitem 17b? *

Copy and paste relevant sections from the manuscript (include quotes in quotation marks "like this" to indicate direct quotes from your manuscript), or elaborate on this item by providing additional information not in the ms, or briefly explain why the item is not applicable/relevant for your study

N/A - No significant effects observed on health status, anxiety and depression, self-efficacy, and most utilization metrics.

18) Results of any other analyses performed, including subgroup analyses and adjusted analyses, distinguishing pre-specified from exploratory

Does your paper address CONSORT subitem 18? *

Copy and paste relevant sections from the manuscript (include quotes in quotation marks "like this" to indicate direct quotes from your manuscript), or elaborate on this item by providing additional information not in the ms, or briefly explain why the item is not applicable/relevant for your study

"Self-care maintenance and physical quality of life improved significantly for HF patients in the telemonitoring group (P= .036 and P=.046, respectively). However, none of the between-group comparisons were statistically significant, likely due to the general improvement in self-care and quality of life scores of the control group and insufficient sample size to detect changes in condition-specific metrics."

18-i) Subgroup analysis of comparing only users

A subgroup analysis of comparing only users is not uncommon in ehealth trials, but if done, it must be stressed that this is a self-selected sample and no longer an unbiased sample from a randomized trial (see 16-iii).

	1	2	3	4	5	
subitem not at all important	○	○	○	○	○	essential

Does your paper address subitem 18-i?

Copy and paste relevant sections from the manuscript (include quotes in quotation marks "like this" to indicate direct quotes from your manuscript), or elaborate on this item by providing additional information not in the ms, or briefly explain why the item is not applicable/relevant for your study

N/A - Subgroup analysis of comparing only users was not conducted.

19) All important harms or unintended effects in each group

(for specific guidance see CONSORT for harms)

Does your paper address CONSORT subitem 19? *

Copy and paste relevant sections from the manuscript (include quotes in quotation marks "like this" to indicate direct quotes from your manuscript), or elaborate on this item by providing additional information not in the ms, or briefly explain why the item is not applicable/relevant for your study

"One death was reported in each treatment arm, however, these were not considered an adverse event of the study."

19-i) Include privacy breaches, technical problems

Include privacy breaches, technical problems. This does not only include physical “harm” to participants, but also incidents such as perceived or real privacy breaches [1], technical problems, and other unexpected/unintended incidents. “Unintended effects” also includes unintended positive effects [2].

	1	2	3	4	5	
subitem not at all important	○	○	○	○	○	essential

Does your paper address subitem 19-i?

Copy and paste relevant sections from the manuscript (include quotes in quotation marks "like this" to indicate direct quotes from your manuscript), or elaborate on this item by providing additional information not in the ms, or briefly explain why the item is not applicable/relevant for your study

There were privacy breaches or technical problems to report

19-ii) Include qualitative feedback from participants or observations from staff/researchers

Include qualitative feedback from participants or observations from staff/researchers, if available, on strengths and shortcomings of the application, especially if they point to unintended/unexpected effects or uses. This includes (if available) reasons for why people did or did not use the application as intended by the developers.

	1	2	3	4	5	
subitem not at all important	○	○	○	○	○	essential

Does your paper address subitem 19-ii?

Copy and paste relevant sections from the manuscript (include quotes in quotation marks "like this" to indicate direct quotes from your manuscript), or elaborate on this item by providing additional information not in the ms, or briefly explain why the item is not applicable/relevant for your study

"Thirteen of the 41 patients who completed the study in the intervention arm were interviewed, including 8 HF patients, 4 HT patients, and 1 DM patient. The HCPs included 1 cardiologist and 2 cardiac nurse practitioners, 1 hypertension specialist, and 1 endocrinologist. The interview findings, which contribute to explaining the overall null results of the study, are summarized in the following themes: (1) challenges of implementing MCC telemonitoring in a siloed healthcare system; (2) perceptions of the telemonitoring system; (3) perceived benefits differed based on the condition monitored; and (4) opportunities for MCC telemonitoring."

DISCUSSION

22) Interpretation consistent with results, balancing benefits and harms, and considering other relevant evidence

NPT: In addition, take into account the choice of the comparator, lack of or partial blinding, and unequal expertise of care providers or centers in each group

22-i) Restate study questions and summarize the answers suggested by the data, starting with primary outcomes and process outcomes (use)

Restate study questions and summarize the answers suggested by the data, starting with primary outcomes and process outcomes (use).

	1	2	3	4	5	
subitem not at all important	○	○	○	○	○	essential

Does your paper address subitem 22-i? *

Copy and paste relevant sections from the manuscript (include quotes in quotation marks "like this" to indicate direct quotes from your manuscript), or elaborate on this item by providing additional information not in the ms, or briefly explain why the item is not applicable/relevant for your study

"In the absence of high-quality evidence on telemonitoring for managing MCCs [47], this study sought to evaluate the impact of a mobile phone based telemonitoring program to manage complex patients in specialty care settings. Through a pragmatic RCT, halted before reaching the intended sample size, we observed no effects of the telemonitoring program on health status, anxiety and depression, self-efficacy, and most utilization metrics. Subgroup analyses of HF patients in the intervention arm found a significant improvement in physical quality of life and self-care maintenance, however, no differences in these groups were observed between treatment arms. The reduction in self-report hospitalization found in the control group could also be seen as a trend in the intervention group but did not reach statistical significance likely due to the small sample size. This reduction for both arms may be partly attributed to the impact of the HF clinic in stabilizing patients."

22-ii) Highlight unanswered new questions, suggest future research

Highlight unanswered new questions, suggest future research.

	1	2	3	4	5	
subitem not at all important	○	○	○	○	○	essential

Does your paper address subitem 22-ii?

Copy and paste relevant sections from the manuscript (include quotes in quotation marks "like this" to indicate direct quotes from your manuscript), or elaborate on this item by providing additional information not in the ms, or briefly explain why the item is not applicable/relevant for your study

"Consistent with previous studies, we recommend that future research conceive telemonitoring as a program, composed of both the system and its associated model of care. Critically, implementation study designs should be used to assess the feasibility of the program prior to conducting a trial, so that challenges may be identified and addressed [50]."

20) Trial limitations, addressing sources of potential bias, imprecision, and, if relevant, multiplicity of analyses

20-i) Typical limitations in ehealth trials

Typical limitations in ehealth trials: Participants in ehealth trials are rarely blinded. Ehealth trials often look at a multiplicity of outcomes, increasing risk for a Type I error. Discuss biases due to non-use of the intervention/usability issues, biases through informed consent procedures, unexpected events.

	1	2	3	4	5	
subitem not at all important	○	○	○	○	○	essential

Does your paper address subitem 20-i? *

Copy and paste relevant sections from the manuscript (include quotes in quotation marks "like this" to indicate direct quotes from your manuscript), or elaborate on this item by providing additional information not in the ms, or briefly explain why the item is not applicable/relevant for your study

"In addition to the recruitment and implementation challenges previously discussed, this study had limitations attributable to the trial's pragmatic nature. First, although the study period was 6 months, the study duration lasted almost 4 years with no overlap in the periods in which HF patients and HT/DM patients were active. This meant that, in addition to inhibiting the exploration of multidisciplinary care coordination, ending recruitment due to COVID-19 contributed to a difference in sample size across conditions. Second, self-reported utilization introduces potential challenges due to recall. Third, unintended selection bias is possible as recruitment relied on busy clinicians identifying eligible patients during clinic hours. We do not have data on patients who might have been eligible but who were never identified by their care team, nor on patients who were approached but did not accept to be introduced to the research coordinator. Finally, the interviews were conducted with a convenience sample of patients, and although all HCPs who were involved in the study in any significant manner were interviewed, the number of five HCPs was relatively small."

21) Generalisability (external validity, applicability) of the trial findings

NPT: External validity of the trial findings according to the intervention, comparators, patients, and care providers or centers involved in the trial

21-i) Generalizability to other populations

Generalizability to other populations: In particular, discuss generalizability to a general Internet population, outside of a RCT setting, and general patient population, including applicability of the study results for other organizations

	1	2	3	4	5	
subitem not at all important	○	○	○	○	○	essential

Does your paper address subitem 21-i?

Copy and paste relevant sections from the manuscript (include quotes in quotation marks "like this" to indicate direct quotes from your manuscript), or elaborate on this item by providing additional information not in the ms, or briefly explain why the item is not applicable/relevant for your study

"Several challenges were encountered during the trial, necessitating deviations from the published protocol, and likely contributed to the null results of the study. Foremost, this trial did not reach its intended sample size of 146. Although the onset of COVID-19 and the rapid shift to virtual care for both arms did bring about the decision to stop recruitment permanently, other important challenges contributed to slow recruitment. Importantly, funding and development challenges contributed to an inability to include the planned chronic obstructive pulmonary disease and chronic kidney disease modules. Similarly, while patients who participated in our study did have MCCs, we were unable to offer monitoring for all the conditions simultaneously because the HF, HT, and DM modules were not initially available at the same time. Therefore, the MCC model of care was never fully tested as there was never a need to explore communication workflows for multidisciplinary care that are inherent to the care of MCCs. Finally, recruitment of patients from the diabetes clinic was challenging due to the emergence of newer continuous glucose monitoring technologies at the time of recruitment."

21-ii) Discuss if there were elements in the RCT that would be different in a routine application setting

Discuss if there were elements in the RCT that would be different in a routine application setting (e.g., prompts/reminders, more human involvement, training sessions or other co-interventions) and what impact the omission of these elements could have on use, adoption, or outcomes if the intervention is applied outside of a RCT setting.

	1	2	3	4	5	
subitem not at all important	○	○	○	○	○	essential

Does your paper address subitem 21-ii?

Copy and paste relevant sections from the manuscript (include quotes in quotation marks "like this" to indicate direct quotes from your manuscript), or elaborate on this item by providing additional information not in the ms, or briefly explain why the item is not applicable/relevant for your study

This was a pragmatic RCT and thus the intervention was applied in a routine application setting.

OTHER INFORMATION

23) Registration number and name of trial registry

Does your paper address CONSORT subitem 23? *

Copy and paste relevant sections from the manuscript (include quotes in quotation marks "like this" to indicate direct quotes from your manuscript), or elaborate on this item by providing additional information not in the ms, or briefly explain why the item is not applicable/relevant for your study

"Trial Registration: ClinicalTrials.gov NCT03127852, ISRCTN (41238563)"

24) Where the full trial protocol can be accessed, if available

Does your paper address CONSORT subitem 24? *

Cite a Multimedia Appendix, other reference, or copy and paste relevant sections from the manuscript (include quotes in quotation marks "like this" to indicate direct quotes from your manuscript), or elaborate on this item by providing additional information not in the ms, or briefly explain why the item is not applicable/relevant for your study

"See the published protocol for full criteria [33]."

25) Sources of funding and other support (such as supply of drugs), role of funders

Does your paper address CONSORT subitem 25? *

Copy and paste relevant sections from the manuscript (include quotes in quotation marks "like this" to indicate direct quotes from your manuscript), or elaborate on this item by providing additional information not in the ms, or briefly explain why the item is not applicable/relevant for your study

"The authors wish to thank the trial participants and the HCPs for both their role in facilitating participant recruitment and in the telemonitoring intervention. This study was funded by the Canadian Institutes for Health Research which had no role in this study."

X27) Conflicts of Interest (not a CONSORT item)

X27-i) State the relation of the study team towards the system being evaluated

In addition to the usual declaration of interests (financial or otherwise), also state the relation of the study team towards the system being evaluated, i.e., state if the authors/evaluators are distinct from or identical with the developers/sponsors of the intervention.

	1	2	3	4	5	
subitem not at all important	○	○	○	○	○	essential

Does your paper address subitem X27-i?

Copy and paste relevant sections from the manuscript (include quotes in quotation marks "like this" to indicate direct quotes from your manuscript), or elaborate on this item by providing additional information not in the ms, or briefly explain why the item is not applicable/relevant for your study

"ES, HR, JC are considered inventors of the Medly system under the intellectual property policies of the UHN and may benefit from future commercialization of the technology by UHN."

About the CONSORT EHEALTH checklist

As a result of using this checklist, did you make changes in your manuscript? *

- ( ) yes, major changes
- (•) yes, minor changes
- ( ) no

What were the most important changes you made as a result of using this checklist?

Your answer

How much time did you spend on going through the checklist INCLUDING making changes in your manuscript *

2 hours to go through the checklist and make changes to the manuscript

As a result of using this checklist, do you think your manuscript has improved? *

- (•) yes
- ( ) no
- ( ) Other:

Would you like to become involved in the CONSORT EHEALTH group?

This would involve for example becoming involved in participating in a workshop and writing an "Explanation and Elaboration" document

- (x) yes
- ( ) no
- ( ) Other:

Clear selection

Any other comments or questions on CONSORT EHEALTH

Your answer

STOP - Save this form as PDF before you click submit

To generate a record that you filled in this form, we recommend to generate a PDF of this page (on a Mac, simply select "print" and then select "print as PDF") before you submit it.

When you submit your (revised) paper to JMIR, please upload the PDF as supplementary file.

Don't worry if some text in the textboxes is cut off, as we still have the complete information in our database. Thank you!

Final step: Click submit !

Click submit so we have your answers in our database!

Submit

Never submit passwords through Google Forms.

This content is neither created nor endorsed by Google. Report Abuse - Terms of Service - Privacy Policy

Google Forms
